# Supplementary material for: Cytotaxonomic characterization and estimation of migration patterns of onchocerciasis vectors (Simulium damnosum sensu lato) in northwestern Ethiopia based on RADSeq data
Source: PLoS Negl Trop Dis. 2024 Jan 4;18(1):e0011868. doi: 10.1371/journal.pntd.0011868 (PMC10793886; doi:10.1371/journal.pntd.0011868)
Supplement: S1 Text — (DOCX) [file pntd.0011868.s001.docx]

## **Preparation and analysis of RADseq data**

A minimum of 100ng DNA was used for RADseq following digestion with *Msl*I. Libraries were sequenced on an Illumina NextSeq 500/550 by LGC Genomics GmbH (Berlin, Germany) using v2 chemistry with 150 bp paired-end reads. Reads were filtered for quality and adapter sequence and the *MsI*I restriction enzyme cut sites removed. Reads <125 bp long were removed using a custom *perl* script. *Stacks* v.2.41 [1] was utilized for a preliminary *de novo* assembly using the lowest parameter values (*M* = *n* = 1) and coverage (average depth across each locus given those parameters) was estimated per sample. Samples with coverage less than 10 were removed from downstream analyses [2].

One of the challenges of RADseq analyses is to determine which parameter values to use when assessing orthology. The number of mismatches (M) allowed between two alleles within a heterozygous individual was set equal to the value used for *n*, the number of mismatches allowed between any two alleles [2]. We used the “r80” approach to optimize the value for mismatches [3]. This approach analyses the data using a range of values for *M* to identify the point at which the number of loci found in 80% of the samples in a population reaches a plateau. Analyses were performed using each sampling location as an independent subpopulation (likely to be an overestimate of the actual number of genetically distinct subpopulations). Based on this analysis, *M* = 4 appeared to be the point at which the number of orthologous loci and the number of variants identified in 80% of the sample reached a plateau (Fig S7). For downstream analyses, the additional conservative approach of limiting the loci used to only those that were found in all sampling locations was taken.

Basic population genetic diversity statistics for each sampling location were estimated using *Stacks* [1]. Two alternative algorithmic approaches for identifying subpopulations of blackflies and computing the posterior probability of each blackfly’s membership in each inferred subpopulation were used: (1) discriminant analysis of principle components (DAPC; [4]) using the R library package *adegenet* v. 2.1.3 [5, 6] and (2) the program *structure* v.2.3.4 for Linux [7].

Principal components (PCs) of genetic variation were plotted using *adegenet*. DAPC was then used to infer the number of clusters or subpopulations suggested by the data using the Bayesian information criterion (BIC) and to assign individuals to those subpopulations. In a separate analysis, maximizing and plotting the differentiation among the six sampling locations was explored. The optimal number of PCs to include in the analysis was determined using cross-validation with 100 replicates, using a training set of 0.9, and a maximum number of 300 PCs.

*Structure* [7] was used to both define the number of sub-populations and to assign individuals to those sub-populations. The sensitivity of the analysis to whether allele frequencies are considered correlated or uncorrelated was tested using two runs with a burn-in of 10,000 and 100,000 post-burn-in generations for both 2 and 6 populations. Because the results were nearly identical in terms of posterior probabilities for population-of-origin (differences less than 0.005), the uncorrelated prior was used as the runs were marginally faster. Stationarity (when parameter values no longer fluctuate across the run) and convergence (similarity of parameter estimates between runs) were assessed by plotting alpha and the log-likelihood per computational generation across each run. Because both stationarity and convergence were reached rapidly, ten replicate runs with a burn-in of 10,000 and 20,000 post-burnin generations were performed for the number of sub-populations (*K*) ranging from 1 to 8 (i.e., 80 runs in total), using the system clock as a seed. Convergence and stationarity were confirmed for each of the ten replicates by plotting both alpha and the log-likelihood. The posterior probability assignment to ancestral populations for each individual fly was averaged across the ten runs for each value of K tested.

Isolation-by-distance occurs when genetic variation of a continuously distributed species with low dispersal range is associated with geographic distance rather than differentiation arising as a result of discontinuous geographic isolation or other types of reproductive isolation. We performed a statistical comparison of geographic distance between sampling sites, estimated using *geosphere* v. 1.5.18 (<https://github.com/rspatial/geosphere>) with pairwise genetic distance [8] and with F_ST_ [9], calculated using the R package *hierfstat* v 0.5.11 [10] (Table S10), using a Monte-Carlo Mantel test [11] with 10 million permutations using *ade4* v 1.7.22 [12]. The result of the permutations indicated that the data were not consistent with an isolation-by-distance model (genetic distance: p = 0.1263583, F_ST_: p = 0.0499657). Note that the fundamental assumption behind this test is that the data are from a single species. For our dataset, we could not ascertain whether the two clusters represented different samples of the same species, or two different species, and thus the biological interpretation of these results is unclear.

1. Catchen J, Hohenlohe PA, Bassham S, Amores A, Cresko WA. Stacks: an analysis tool set for population genomics. Mol Ecol. 2013;22(11):3124-40. Epub 2013/05/25. doi: 10.1111/mec.12354. PubMed PMID: 23701397; PubMed Central PMCID: PMCPMC3936987.

2. Rochette NC, Catchen JM. Deriving genotypes from RAD-seq short-read data using Stacks. Nat Protoc. 2017;12(12):2640-59. Epub 2017/12/01. doi: 10.1038/nprot.2017.123. PubMed PMID: 29189774.

3. Paris JR, Stevens JR, Catchen JM. Lost in parameter space: a road map for STACKS. Methods Ecol Evol. 2017;8(10):1360-73. doi: 10.1111/2041-210X.12775.

4. Jombart T, Devillard S, Balloux F. Discriminant analysis of principal components: a new method for the analysis of genetically structured populations. BMC Genet. 2010;11:94. doi: 10.1186/1471-2156-11-94. PubMed PMID: 20950446; PubMed Central PMCID: PMCPMC2973851.

5. Jombart T. adegenet: a R package for the multivariate analysis of genetic markers. Bioinformatics. 2008;24(11):1403-5. doi: 10.1093/bioinformatics/btn129. PubMed PMID: 18397895.

6. Jombart T, Ahmed I. adegenet 1.3-1: new tools for the analysis of genome-wide SNP data. Bioinformatics. 2011;27(21):3070-1. doi: 10.1093/bioinformatics/btr521. PubMed PMID: 21926124; PubMed Central PMCID: PMCPMC3198581.

7. Pritchard JK, Stephens M, Donnelly P. Inference of population structure using multilocus genotype data. Genetics. 2000;155(2):945-59. Epub 2000/06/03. doi: 10.1093/genetics/155.2.945. PubMed PMID: 10835412; PubMed Central PMCID: PMCPMC1461096.

8. Takezaki N, Nei M. Genetic distances and reconstruction of phylogenetic trees from microsatellite DNA. Genetics. 1996;144(1):389-99. doi: 10.1093/genetics/144.1.389. PubMed PMID: 8878702; PubMed Central PMCID: PMCPMC1207511.

9. Weir BS, Cockerham CC. Estimating F-statistics for the analysis of population structure. Evolution. 1984;38(6):1358-70.

10. Goudet J. HIERFSTAT, a package for R to compute and test hierarchical *F*-statistics. Mol Ecol Notes. 2005;5:184-6. doi: 10.1111/j.1471-8278 .2004.00828.

11. Mantel N. The detection of disease clustering and a generalized regression approach. Cancer Res. 1967;27(2):209-20. PubMed PMID: 6018555.

12. Dray S, Dufour A. The ade4 package: implementing the duality diagram for ecologists. J Stat Softw. 2007;22(4):1-20. doi: 10.18637/jss.v022.i04.
