# Supplementary material for: Cytotaxonomic characterization and estimation of migration patterns of onchocerciasis vectors (Simulium damnosum sensu lato) in northwestern Ethiopia based on RADSeq data
Source: PLoS Negl Trop Dis. 2024 Jan 4;18(1):e0011868. doi: 10.1371/journal.pntd.0011868 (PMC10793886; doi:10.1371/journal.pntd.0011868)
Supplement: S1 Table — (DOCX) [file pntd.0011868.s002.docx]

### **Table S1.** List of *Simulium* species recorded as anthropophilic in Ethiopia (after Mehbrahtu *et al*. 1980).

| **Subgenus** | ***Simulium* Species** | **Onchocerciasis Vector Status in Ethiopia** |
| --- | --- | --- |
| *Edwardsellum* | *S. damnosum* s.l. | Species complex, with some vectors and some non-anthropophilic |
| *Lewisellum* | *S. ethiopiense* | Vector |
| *Lewisellum* | (*S. nyasalandicum*) | Almost certainly a historical misidentification, not anthropophilic in other countries |
| *Anasolen* | *S. dentulosum* | Probable non-vector |
| *Anasolen* | *S. shoae* | Probable non-vector |
| *Meilloniellum* | *S. adersi* | Probable non-vector |
| *Metomphallus* | *S. gibense* | Probable non-vector |
| *Metomphallus* | *S. bovis* | Not recognised as anthropophilic by Mebrahtu [1], but collected by human landing catches in this study. |

##

Mehbrahtu Y, Abebe M, Mekuria Y. Blackflies (Diptera: Simuliidae) of Ethiopia: checklist and distribution. SINET Ethiopian J Sci. 1980;3:1-20.
