# Supplementary material for: Cytotaxonomic characterization and estimation of migration patterns of onchocerciasis vectors (Simulium damnosum sensu lato) in northwestern Ethiopia based on RADSeq data
Source: PLoS Negl Trop Dis. 2024 Jan 4;18(1):e0011868. doi: 10.1371/journal.pntd.0011868 (PMC10793886; doi:10.1371/journal.pntd.0011868)
Supplement: S2 Table — (DOCX) [file pntd.0011868.s003.docx]

### **Table S2.** Sample sites in northwestern Ethiopia where larvae or biting adult female *Simulium* sp. were collected.

| **Site** | **Transmission zone** | **Locality** | **Zone** | **Woreda** | **Kebele** | **River** | **Life stage** | **Number +*S. bovis*** | **Date of collection** | **Lat** | **Long** | **Altitude** |
| --- | --- | --- | --- | --- | --- | --- | --- | --- | --- | --- | --- | --- |
| 1 | Metema | Wudi Gemzu | North Gondar | Metema | Diviko | Wudi Gemzu | larvae | 10 | 10-Oct-13 | 13.00639 | 36.28278 | 719 |
| 2 | Metema | Guange N^o^ 6 | North Gondar | Metema |  | Kibe | larvae | 3 | 10-Oct-13 | 12.91778 | 36.25222 | 692 |
| 3 | Metema | Meka | North Gondar | Metema |  | Meka | larvae | 2 | 17-Oct-13 | 12.68667 | 36.51528 | 794 |
| 4 | Metema | Dubaba Kebele | North Gondar | Metema |  | Guangie | larvae | 17 | 14-Oct-13 | 12.4225 | 36.93917 | 613 |
| 5 | Metema | Delegu Anana | North Gondar | Metema |  | Delegu Namo | larvae | 4 | 14-Oct-13 | 12.21639 | 35.90167 | 661 |
| A | Metekel | Selassie Godiguadit | North Gondar | Alefa | Genet Guancha | Tana Beles | adult f | 33+0 | April to June 2017 | 11.75194 | 36.84825 |  |
| B | Metekel | Block 4 | Awi | Jawi | Alkurand/Block 4 | Abat Beles | adult f | 33+0 | Jan to Dec 2017 | 11.53691 | 36.54976 |  |
| C | Metema | Asakefari | North Gondar | Metema | Diviko | Asakefari | adult f | 15+16 | June to August 2018 | 12.87524 | 36.40832 |  |
| D | Metema | Wudi Gemzu | North Gondar | Metema | Diviko | Wudi Gemzu | adult f | 29+2 | May to August 2018 | 13.00668 | 36.28284 |  |
| E | Metema | Kisha | North Gondar | Tsegedie | Ergoye | Angareb | adult f | 6+23 | July 2017 to April 2018 | 13.24954 | 36.79217 |  |
| F | Metema | Nega Wuha | North Gondar | Metema | Wudi Ambeso | Wudi Gemzu | adult f | 32+0 | July to August 2018 | 13.02625 | 36.43892 |  |
