# Supplementary material for: Cytotaxonomic characterization and estimation of migration patterns of onchocerciasis vectors (Simulium damnosum sensu lato) in northwestern Ethiopia based on RADSeq data
Source: PLoS Negl Trop Dis. 2024 Jan 4;18(1):e0011868. doi: 10.1371/journal.pntd.0011868 (PMC10793886; doi:10.1371/journal.pntd.0011868)
Supplement: S3 Table — (DOCX) [file pntd.0011868.s004.docx]

### **Table S3.** Inversion Frequencies in the *Simulium damnosum* subcomplex from Ethiopia. m = male; f = female; nd = not determined.

| **Sample-River** | **Number** | **1S** | | | **1L** | | | | | **2L** | | | | **3L** | |
| --- | --- | --- | --- | --- | --- | --- | --- | --- | --- | --- | --- | --- | --- | --- | --- |
|  |  | **1** | **2** | **3** | **1** | **3** | **22*** | **23*** | **24*** | **C** | **8** | **2b*** | **70*** | **2** | **6** |
|  |  |  |  |  |  |  |  |  |  |  |  |  |  |  |  |
| Wodigemzu | 10 | 1.00 | 0.43 | 0.58 | 0.86 | 1.00 | 0.17 | 0.17 | 0 | 1.00 | 0.63 | 0.38 | 0 | 1.00 | 1.00 |
| Kibe | 3 | 1.00 | 0.50 | 0.50 | 1.00 | 1.00 | 0 | 0 | 0 | 1.00 | 0.50 | 0.50 | 0 | 1.00 | 1.00 |
| Meka | 2 | 1.00 | 0.75 | 1.00 | 1.00 | 1.00 | 0 | 0 | 0 | 1.00 | 1.00 | 0 | 0 | 1.00 | 1.00 |
| Guangie | 17 | 1.00 | 0.71 | 0.91 | 1.00 | 1.00 | 0.06 | 0.15 | 0.09 | 1.00 | 0.79 | 0.21 | 0.03 | 1.00 | 1.00 |
| Delegu Namo | 4 | 1.00 | 1.00 | 1.00 | 1.00 | 1.00 | 0 | 0 | 0 | 1.00 | 1.00 | 0 | 0 | 1.00 | 1.00 |

*New inversions unknown outside Ethiopia
