# Supplementary material for: Cytotaxonomic characterization and estimation of migration patterns of onchocerciasis vectors (Simulium damnosum sensu lato) in northwestern Ethiopia based on RADSeq data
Source: PLoS Negl Trop Dis. 2024 Jan 4;18(1):e0011868. doi: 10.1371/journal.pntd.0011868 (PMC10793886; doi:10.1371/journal.pntd.0011868)
Supplement: S4 Table — (DOCX) [file pntd.0011868.s005.docx]

### **Table S4.** Karyotype distribution of *Simulium damnosum* subcomplex from Ethiopia: Chromosome 2. m = male; f = female; nd = not determined.

| **River** | **Number** | ***S. damnosum* *sensu stricto* (?)** | | | | ***S. sirbanum* (?)** | | |
| --- | --- | --- | --- | --- | --- | --- | --- | --- |
|  |  | **2L-C/8.2b** | **2L-C/8.2b** | **2L-C.2b** | **2L-C.2b** | **2L-C.8** | **2L-C.8** | **2L-C.8** |
|  |  | **♂** | **♀** | **♂** | **♀** | **♂** | **♀** | nd |
| Wodigemzu | 1 m, 6 f, 1 nd |  | 2 |  | 2 | 1 | 2 |  |
| Kibe | 1 m, 2 f | 1 |  |  | 1 |  | 1 |  |
| Meka | 2 m |  |  |  |  | 2 |  |  |
| Guangie | 10 m, 7 f | 2 | 1 |  | 2 | 8 | 2 (+2) |  |
| Delegu | 3 m, 1 nd |  |  |  |  | 3 |  | 1 |
| **Total** | **17 m, 15 f, 2 nd** | **3** | **3** | **0** | **5** | **14** | **7** | **1** |
