# Supplementary material for: Cytotaxonomic characterization and estimation of migration patterns of onchocerciasis vectors (Simulium damnosum sensu lato) in northwestern Ethiopia based on RADSeq data
Source: PLoS Negl Trop Dis. 2024 Jan 4;18(1):e0011868. doi: 10.1371/journal.pntd.0011868 (PMC10793886; doi:10.1371/journal.pntd.0011868)
Supplement: S5 Table — (DOCX) [file pntd.0011868.s006.docx]

### **Table S5.** Karyotype distribution of *S. damnosum* subcomplex from Ethiopia: Chromosome 1L. m = male; f = female; nd = not determined.

| **River** | **Number** | **IL/1** | **IL/1** | **IL-1** | **IL-1** | **IL st** | **IL st** | **IL/3** | **IL/3** | **IL-3** | **IL-3** | **IL st** | **IL st** |
| --- | --- | --- | --- | --- | --- | --- | --- | --- | --- | --- | --- | --- | --- |
|  |  | **♂** | **♀** | **♂** | **♀** | **♂** | **♀** | **♂** | **♀** | **♂** | **♀** | **♂** | **♀** |
| Wodigemzu | 1 m, 6 fem, 1 nd |  | 2 |  | 5 |  |  |  |  | 1 | 5 |  |  |
| Kibe | 1 m, 2 fem |  |  | 1 | 2 |  |  |  |  | 1 | 2 |  |  |
| Meka | 2 m |  |  | 2 |  |  |  |  |  | 2 |  |  |  |
| Guangie | 10 m, 7 fem |  | 1 | 10 | 5 |  | 1 | 2 | 1 | 8 | 6 |  |  |
| Delegu | 3 m, 1 nd |  |  | 3 |  |  |  |  |  | 3 |  |  |  |
| **Total** | **17 m, 15 fem, 2 nd** | **0** | **3** | **16** | **12** | **0** | **1** | **2** | **1** | **15** | **13** | **0** | **0** |
