# Supplementary material for: Cytotaxonomic characterization and estimation of migration patterns of onchocerciasis vectors (Simulium damnosum sensu lato) in northwestern Ethiopia based on RADSeq data
Source: PLoS Negl Trop Dis. 2024 Jan 4;18(1):e0011868. doi: 10.1371/journal.pntd.0011868 (PMC10793886; doi:10.1371/journal.pntd.0011868)
Supplement: S6 Table — (DOCX) [file pntd.0011868.s007.docx]

### **Table S6.** Karyotype distribution of *S. damnosum* subcomplex from Ethiopia: Chromosome 1S. m = male; f = female; nd = not determined.

| **River** | **Number** | **IS/2** | **IS/2** | **IS-2** | **IS-2** | **IS st** | **IS st** | **IS/3** | **IS/3** | **IS-3** | **IS-3** | **IS st** | **IS st** |
| --- | --- | --- | --- | --- | --- | --- | --- | --- | --- | --- | --- | --- | --- |
|  |  | **♂** | **♀** | **♂** | **♀** | **♂** | **♀** | **♂** | **♀** | **♂** | **♀** | **♂** | **♀** |
| Wodigemzu | 1 m, 6 f, 1 nd | 1 | 3 |  | 1 |  | 1 |  | 3 | 1 | 1 |  | 1 |
| Kibe | 1 m, 2 f | 1 |  |  | 1 |  | 1 |  | 1 |  | 1 |  | 1 |
| Meka | 2 m | 1 | 1 |  |  |  |  |  |  | 2 |  |  |  |
| Guangie | 10 m, 7 f | 5 | 1 | 5 | 4 |  | 2 | 1 | 1 | 9 | 4 |  | 2 |
| Delegu | 3 m, 1 nd |  |  | 3 |  |  |  |  |  | 3 |  |  |  |
| **Total** | **17 m, 15 f, 2 nd** | **8** | **5** | **8** | **6** | **0** | **4** | **1** | **5** | **15** | **6** | **0** | **4** |
