# Supplementary material for: Cytotaxonomic characterization and estimation of migration patterns of onchocerciasis vectors (Simulium damnosum sensu lato) in northwestern Ethiopia based on RADSeq data
Source: PLoS Negl Trop Dis. 2024 Jan 4;18(1):e0011868. doi: 10.1371/journal.pntd.0011868 (PMC10793886; doi:10.1371/journal.pntd.0011868)
Supplement: S7 Table — (DOCX) [file pntd.0011868.s008.docx]

### **Table S7.** Genetic diversity statistics across species and populations of *Simulium damnosum s.l., S. bovis*, and the query-*damnosum* samples based on CoI DNA sequence data. Statistics reported are sample size (n), number of haplotypes (H), number of polymorphic (segregating) sites (S), average number of nucleotide differences (K), nucleotide diversity (π*_n_*), haplotype diversity (π*_h_*), Tajima’s *D*, and *F*_S_. Collection sites indicated are defined in Table S2. * : statistically significant at p < 0.05; **: statistically significant at p < 0.01.

| Population | *n* | H | S | K | π*_n_* (SD) | π*_h_* (SD) | Tajima’s *D* | *F*_S_ |
| --- | --- | --- | --- | --- | --- | --- | --- | --- |
| All samples | 189 | 102 | 127 | 31.234 | 0.048 (0.004) | 0.935 (0.014) | 0.406 | -23.37 |
|  |  |  |  |  |  |  |  |  |
| All *S. damnosum* | 142 | 55 | 55 | 2.978 | 0.005 (0.001) | 0.885 (0.022) | -2.236** | -63.544 |
| *S. damnosum* A | 31 | 17 | 35 | 3.318 | 0.005 (0.002) | 0.826 (0.068) | -2.299** | -8.524 |
| *S. damnosum* B | 29 | 15 | 27 | 3.53 | 0.005 (0.046) | 0.879 (0.046) | -1.826* | -5.755 |
| *S. damnosum* C | 15 | 10 | 21 | 3.124 | 0.005 (0.002) | 0.895 (0.07) | -2.123** | -3.965 |
| *S. damnosum* D | 29 | 16 | 23 | 3.128 | 0.005 (0.001) | 0.914 (0.035) | -1.822* | -8.088 |
| *S. damnosum* E | 6 | 5 | 6 | 2.2 | 0.003 (0.001) | 0.933 (0.122) | -0.932 | -1.909 |
| *S. damnosum* F | 32 | 19 | 19 | 2.109 | 0.003 (0.000) | 0.927 (0.034) | -1.892* | -16.843 |
|  |  |  |  |  |  |  |  |  |
| All query *S. damnosum* | 6 | 6 | 17 | 7.4 | 0.011 (0.002) | 1 (0.096) | -0.038 | -1.443 |
| Query *damnosum* A | 2 | 2 | 4 | 4 | 0.006 (0.003) | 1 (0.5) | N/A | N/A |
| Query *damnosum* B | 4 | 4 | 14 | 7.167 | 0.011 (0.003) | 1 (0.177) | -0.624 | -0.065 |
|  |  |  |  |  |  |  |  |  |
| All *S. bovis* | 41 | 41 | 57 | 7.327 | 0.011 (0.005) | 1 (0.005) | -1.677 | -33.237 |
| *S. bovis* C | 16 | 16 | 41 | 8.542 | 0.013 (0.001) | 1 (0.022) | -1.435 | -9.368 |
| *S. bovis* D | 2 | 2 | 6 | 6 | 0.009 (0.005) | 1 (0.5) | N/A | N/A |
| *S. bovis* E | 23 | 23 | 40 | 6.64 | 0.010 (0.001) | 1 (0.013) | -1.499 | -20.583 |
