# Supplementary material for: Cytotaxonomic characterization and estimation of migration patterns of onchocerciasis vectors (Simulium damnosum sensu lato) in northwestern Ethiopia based on RADSeq data
Source: PLoS Negl Trop Dis. 2024 Jan 4;18(1):e0011868. doi: 10.1371/journal.pntd.0011868 (PMC10793886; doi:10.1371/journal.pntd.0011868)
Supplement: S8 Table — (DOCX) [file pntd.0011868.s009.docx]

### **Table S8**. Sequencing statistics for RADSeq data based on STACKS when M = 1. Samples with mean coverage < 10 were removed.

| **Sample ID** | **Site Code** | **Location** | **Mean coverage (M = 1)** | **Standard deviation** | **Max** | **Number of reads** |  |  |  |  |  |  |  |
| --- | --- | --- | --- | --- | --- | --- | --- | --- | --- | --- | --- | --- | --- |
| SIM-1 | A | N.Gondar,Alefa,Genet Guancha Silasie Godguadit, Tana Beles | 34.63 | 28.7 | 236 | 1767562(88.6%) |  |  |  |  |  |  |  |
| SIM-2 | A | N.Gondar,Alefa,Genet Guancha Silasie Godguadit, Tana Beles | 25.94 | 21.34 | 176 | 1231263(86.7%) |  |  |  |  |  |  |  |
| SIM-3 | B | Awi,Jawi,Block 4,Block 4, Abat Beles | 29.77 | 23.64 | 196 | 1451174(89.4%) |  |  |  |  |  |  |  |
| SIM-4 | B | Awi,Jawi,Block 4,Block 4, Abat Beles | 30.97 | 27.44 | 216 | 1773793(82.3%) |  |  |  |  |  |  |  |
| SIM-5 | C | N.Gondar,Metema,Diviko,Asafekari,Guang | 25.84 | 19.98 | 172 | 1222097(88.6%) |  |  |  |  |  |  |  |
| SIM-7 | D | N.Gondar,Metema,Diviko,Wudi Gemzu,Wudi Gemzu | 29.39 | 22.8 | 203 | 1508040(88.1%) |  |  |  |  |  |  |  |
| SIM-8 | D | N.Gondar,Metema,Diviko,Wudi Gemzu,Wudi Gemzu | 18.3 | 13.53 | 112 | 857054(85.6%) |  |  |  |  |  |  |  |
| SIM-11 | F | N.Gondar,Metema,Wudie Ambeso, Nega Wuha, Wudi Gemzu | 12.37 | 8.14 | 69 | 483651(87.1%) |  |  |  |  |  |  |  |
| SIM-12 | F | N.Gondar,Metema,Wudie Ambeso, Nega Wuha, Wudi Gemzu | 31.54 | 24.89 | 274 | 1497843(88.5%) |  |  |  |  |  |  |  |
| SIM-14 | A | N.Gondar,Alefa,Genet Guancha Silasie Godguadit, Tana Beles | 52.65 | 44.17 | 348 | 2785546(88.2%) |  |  |  |  |  |  |  |
| SIM-15 | A | N.Gondar,Alefa,Genet Guancha Silasie Godguadit, Tana Beles | 38.2 | 30.39 | 258 | 1852773(89.4%) |  |  |  |  |  |  |  |
| SIM-16 | B | Awi,Jawi,Block 4,Block 4, Abat Beles | 34.62 | 29.36 | 229 | 1675516(88.5%) |  |  |  |  |  |  |  |
| SIM-17 | B | Awi,Jawi,Block 4,Block 4, Abat Beles | 35.6 | 28.58 | 240 | 1770823(88.8%) |  |  |  |  |  |  |  |
| SIM-18 | B | Awi,Jawi,Block 4,Block 4, Abat Beles | 27.23 | 22.22 | 182 | 1294528(87.1%) |  |  |  |  |  |  |  |
| SIM-19 | C | N.Gondar,Metema,Diviko,Asafekari,Guang | 8.64 | 5.04 | 47 | 289359(83.5%) |  |  |  |  |  |  |  |
| SIM-21 | C | N.Gondar,Metema,Diviko,Asafekari,Guang | 9.93 | 6.13 | 60 | 347637(85.9%) |  |  |  |  |  |  |  |
| SIM-22 | D | N.Gondar,Metema,Diviko,Wudi Gemzu,Wudi Gemzu | 18.5 | 13.86 | 118 | 801526(87.7%) |  |  |  |  |  |  |  |
| SIM-23 | D | N.Gondar,Metema,Diviko,Wudi Gemzu,Wudi Gemzu | 16.35 | 14.8 | 146 | 756175(72.8%) |  |  |  |  |  |  |  |
| SIM-24 | D | N.Gondar,Metema,Diviko,Wudi Gemzu,Wudi Gemzu | 20.46 | 14.8 | 131 | 904090(88.4%) |  |  |  |  |  |  |  |
| SIM-28 | F | N.Gondar,Metema,Wudie Ambeso, Nega Wuha, Wudi Gemzu | 27.73 | 24.33 | 207 | 1284124(85.9%) |  |  |  |  |  |  |  |
| SIM-29 | F | N.Gondar,Metema,Wudie Ambeso, Nega Wuha, Wudi Gemzu | 18.7 | 16.57 | 144 | 750152(84.1%) |  |  |  |  |  |  |  |
| SIM-30 | A | N.Gondar,Alefa,Genet Guancha Silasie Godguadit, Tana Beles | 19.91 | 17.39 | 167 | 814976(85.0%) |  |  |  |  |  |  |  |
| SIM-31 | A | N.Gondar,Alefa,Genet Guancha Silasie Godguadit, Tana Beles | 21.84 | 18.57 | 159 | 943403(86.1%) |  |  |  |  |  |  |  |
| SIM-32 | A | N.Gondar,Alefa,Genet Guancha Silasie Godguadit, Tana Beles | 25.87 | 21.54 | 179 | 1160007(88.2%) |  |  |  |  |  |  |  |
| SIM-33 | B | Awi,Jawi,Block 4,Block 4, Abat Beles | 30.78 | 26.14 | 223 | 1521680(91.0%) |  |  |  |  |  |  |  |
| SIM-34 | B | Awi,Jawi,Block 4,Block 4, Abat Beles | 15.46 | 13.12 | 114 | 605359(79.1%) |  |  |  |  |  |  |  |
| SIM-35 | B | Awi,Jawi,Block 4,Block 4, Abat Beles | 10.94 | 8.91 | 83 | 315525(80.0%) |  |  |  |  |  |  |  |
| SIM-37 | C | N.Gondar,Metema,Diviko,Asafekari,Guang | 8.72 | 5.59 | 50 | 236564(81.0%) |  |  |  |  |  |  |  |
| SIM-38 | C | N.Gondar,Metema,Diviko,Asafekari,Guang | 10.2 | 6.77 | 64 | 346689(84.6%) |  |  |  |  |  |  |  |
| SIM-39 | D | N.Gondar,Metema,Diviko,Wudi Gemzu,Wudi Gemzu | 14.69 | 10.67 | 105 | 596198(86.8%) |  |  |  |  |  |  |  |
| SIM-40 | D | N.Gondar,Metema,Diviko,Wudi Gemzu,Wudi Gemzu | 12.45 | 8.88 | 99 | 510551(85.0%) |  |  |  |  |  |  |  |
| SIM-41 | D | N.Gondar,Metema,Diviko,Wudi Gemzu,Wudi Gemzu | 8.41 | 5 | 57 | 254637(82.5%) |  |  |  |  |  |  |  |
| SIM-43 | E | N.Gondar, Tsegedie, Ergoye, Kisha, Angereb | 15 | 10.46 | 91 | 633754(86.8%) |  |  |  |  |  |  |  |
| SIM-46 | F | N.Gondar,Metema,Wudie Ambeso, Nega Wuha, Wudi Gemzu | 10.57 | 7.34 | 61 | 352549(84.8%) |  |  |  |  |  |  |  |
| SIM-47 | F | N.Gondar,Metema,Wudie Ambeso, Nega Wuha, Wudi Gemzu | 19.88 | 14.73 | 128 | 872379(88.6%) |  |  |  |  |  |  |  |
| SIM-48 | F | N.Gondar,Metema,Wudie Ambeso, Nega Wuha, Wudi Gemzu | 11.58 | 9.52 | 88 | 349066(81.5%) |  |  |  |  |  |  |  |
| SIM-49 | A | N.Gondar,Alefa,Genet Guancha Silasie Godguadit, Tana Beles | 20.59 | 16.42 | 134 | 890758(87.8%) |  |  |  |  |  |  |  |
| SIM-50 | A | N.Gondar,Alefa,Genet Guancha Silasie Godguadit, Tana Beles | 16.51 | 13.54 | 125 | 661575(84.9%) |  |  |  |  |  |  |  |
| SIM-51 | A | N.Gondar,Alefa,Genet Guancha Silasie Godguadit, Tana Beles | 28.07 | 24.5 | 205 | 1296551(85.9%) |  |  |  |  |  |  |  |
| SIM-52 | B | Awi,Jawi,Block 4,Block 4, Abat Beles | 43.64 | 38.99 | 318 | 2256656(87.7%) |  |  |  |  |  |  |  |
| SIM-53 | B | Awi,Jawi,Block 4,Block 4, Abat Beles | 9.01 | 5.5 | 55 | 295554(82.9%) |  |  |  |  |  |  |  |
| SIM-54 | B | Awi,Jawi,Block 4,Block 4, Abat Beles | 20.65 | 19.07 | 172 | 860445(83.4%) |  |  |  |  |  |  |  |
| SIM-55 | C | N.Gondar,Metema,Diviko,Asafekari,Guang | 10.23 | 7.04 | 64 | 332295(83.2%) |  |  |  |  |  |  |  |
| SIM-56 | C | N.Gondar,Metema,Diviko,Asafekari,Guang | 22.46 | 19.3 | 175 | 1009632(85.7%) |  |  |  |  |  |  |  |
| SIM-58 | D | N.Gondar,Metema,Diviko,Wudi Gemzu,Wudi Gemzu | 19.72 | 17.43 | 168 | 874293(83.9%) |  |  |  |  |  |  |  |
| SIM-59 | D | N.Gondar,Metema,Diviko,Wudi Gemzu,Wudi Gemzu | 9.56 | 6.13 | 56 | 331512(82.4%) |  |  |  |  |  |  |  |
| SIM-60 | D | N.Gondar,Metema,Diviko,Wudi Gemzu,Wudi Gemzu | 23.69 | 21.09 | 170 | 1294097(82.9%) |  |  |  |  |  |  |  |
| SIM-64 | F | N.Gondar,Metema,Wudie Ambeso, Nega Wuha, Wudi Gemzu | 9.46 | 6.21 | 53 | 282571(82.6%) |  |  |  |  |  |  |  |
| SIM-66 | F | N.Gondar,Metema,Wudie Ambeso, Nega Wuha, Wudi Gemzu | 11.32 | 7.71 | 70 | 407455(84.7%) |  |  |  |  |  |  |  |
| SIM-67 | A | N.Gondar,Alefa,Genet Guancha Silasie Godguadit, Tana Beles | 12.48 | 9.59 | 98 | 499035(78.1%) |  |  |  |  |  |  |  |
| SIM-68 | A | N.Gondar,Alefa,Genet Guancha Silasie Godguadit, Tana Beles | 9.9 | 6.52 | 57 | 320921(83.7%) |  |  |  |  |  |  |  |
| SIM-69 | A | N.Gondar,Alefa,Genet Guancha Silasie Godguadit, Tana Beles | 17.49 | 12.79 | 131 | 769344(86.0%) |  |  |  |  |  |  |  |
| SIM-70 | B | Awi,Jawi,Block 4,Block 4, Abat Beles | 25.79 | 19.81 | 153 | 1283077(88.2%) |  |  |  |  |  |  |  |
| SIM-71 | B | Awi,Jawi,Block 4,Block 4, Abat Beles | 15.48 | 10.83 | 113 | 641177(87.5%) |  |  |  |  |  |  |  |
| SIM-72 | B | Awi,Jawi,Block 4,Block 4, Abat Beles | 11.89 | 7.98 | 71 | 472763(83.9%) |  |  |  |  |  |  |  |
| SIM-76 | D | N.Gondar,Metema,Diviko,Wudi Gemzu,Wudi Gemzu | 12.87 | 9.17 | 75 | 562845(81.0%) |  |  |  |  |  |  |  |
| SIM-77 | D | N.Gondar,Metema,Diviko,Wudi Gemzu,Wudi Gemzu | 14.52 | 10.99 | 95 | 658389(83.0%) |  |  |  |  |  |  |  |
| SIM-78 | D | N.Gondar,Metema,Diviko,Wudi Gemzu,Wudi Gemzu | 27.91 | 25.88 | 221 | 1362882(84.5%) |  |  |  |  |  |  |  |
| SIM-79 | E | N.Gondar, Tsegedie, Ergoye, Kisha, Angereb | 42.22 | 37.09 | 304 | 2234547(85.9%) |  |  |  |  |  |  |  |
| SIM-82 | F | N.Gondar,Metema,Wudie Ambeso, Nega Wuha, Wudi Gemzu | 12.19 | 8.29 | 76 | 459675(85.6%) |  |  |  |  |  |  |  |
| SIM-83 | F | N.Gondar,Metema,Wudie Ambeso, Nega Wuha, Wudi Gemzu | 13.59 | 10.09 | 92 | 506222(86.4%) |  |  |  |  |  |  |  |
| SIM-84 | F | N.Gondar,Metema,Wudie Ambeso, Nega Wuha, Wudi Gemzu | 13.78 | 9.74 | 84 | 544353(87.0%) |  |  |  |  |  |  |  |
| SIM-85 | A | N.Gondar,Alefa,Genet Guancha Silasie Godguadit, Tana Beles | 14.69 | 10.01 | 87 | 619245(88.5%) |  |  |  |  |  |  |  |
| SIM-86 | A | N.Gondar,Alefa,Genet Guancha Silasie Godguadit, Tana Beles | 21.95 | 17.85 | 158 | 988534(87.4%) |  |  |  |  |  |  |  |
| SIM-87 | A | N.Gondar,Alefa,Genet Guancha Silasie Godguadit, Tana Beles | 13.45 | 9.64 | 88 | 528362(86.6%) |  |  |  |  |  |  |  |
| SIM-88 | B | Awi,Jawi,Block 4,Block 4, Abat Beles | 15.85 | 11.48 | 99 | 649519(88.0%) |  |  |  |  |  |  |  |
| SIM-89 | B | Awi,Jawi,Block 4,Block 4, Abat Beles | 22.04 | 16.73 | 141 | 997445(88.1%) |  |  |  |  |  |  |  |
| SIM-90 | B | Awi,Jawi,Block 4,Block 4, Abat Beles | 11.21 | 7.55 | 74 | 405801(85.8%) |  |  |  |  |  |  |  |
| SIM-93 | C | N.Gondar,Metema,Diviko,Asafekari,Guang | 14.5 | 11.27 | 159 | 713047(84.6%) |  |  |  |  |  |  |  |
| SIM-95 | D | N.Gondar,Metema,Diviko,Wudi Gemzu,Wudi Gemzu | 7.5 | 4.54 | 47 | 167112(75.1%) |  |  |  |  |  |  |  |
| SIM-97 | E | N.Gondar, Tsegedie, Ergoye, Kisha, Angereb | 10.67 | 6.92 | 78 | 378925(84.2%) |  |  |  |  |  |  |  |
| SIM-100 | F | N.Gondar,Metema,Wudie Ambeso, Nega Wuha, Wudi Gemzu | 18.55 | 13.72 | 127 | 846456(87.8%) |  |  |  |  |  |  |  |
| SIM-101 | F | N.Gondar,Metema,Wudie Ambeso, Nega Wuha, Wudi Gemzu | 13.1 | 8.98 | 77 | 536192(85.7%) |  |  |  |  |  |  |  |
| SIM-103 | A | N.Gondar,Alefa,Genet Guancha Silasie Godguadit, Tana Beles | 11.35 | 7.94 | 73 | 412199(84.9%) |  |  |  |  |  |  |  |
| SIM-104 | A | N.Gondar,Alefa,Genet Guancha Silasie Godguadit, Tana Beles | 26.65 | 25.8 | 226 | 1263348(83.1%) |  |  |  |  |  |  |  |
| SIM-105 | A | N.Gondar,Alefa,Genet Guancha Silasie Godguadit, Tana Beles | 14.26 | 10.8 | 93 | 615886(86.5%) |  |  |  |  |  |  |  |
| SIM-106 | B | Awi,Jawi,Block 4,Block 4, Abat Beles | 9.46 | 6.32 | 146 | 315295(74.0%) |  |  |  |  |  |  |  |
| SIM-107 | B | Awi,Jawi,Block 4,Block 4, Abat Beles | 16.03 | 12.16 | 109 | 710850(87.0%) |  |  |  |  |  |  |  |
| SIM-108 | B | Awi,Jawi,Block 4,Block 4, Abat Beles | 17.71 | 13.2 | 113 | 821781(87.1%) |  |  |  |  |  |  |  |
| SIM-111 | C | N.Gondar,Metema,Diviko,Asafekari,Guang | 8.73 | 5.56 | 56 | 310927(76.1%) |  |  |  |  |  |  |  |
| SIM-114 | D | N.Gondar,Metema,Diviko,Wudi Gemzu,Wudi Gemzu | 13.38 | 9.55 | 99 | 623254(85.8%) |  |  |  |  |  |  |  |
| SIM-116 | E | N.Gondar, Tsegedie, Ergoye, Kisha, Angereb | 61.23 | 102.63 | 795 | 2158941(61.0%) |  |  |  |  |  |  |  |
| SIM-118 | F | N.Gondar,Metema,Wudie Ambeso, Nega Wuha, Wudi Gemzu | 13.93 | 10.01 | 89 | 603579(86.7%) |  |  |  |  |  |  |  |
| SIM-119 | F | N.Gondar,Metema,Wudie Ambeso, Nega Wuha, Wudi Gemzu | 16.07 | 12.56 | 126 | 693871(86.1%) |  |  |  |  |  |  |  |
| SIM-120 | F | N.Gondar,Metema,Wudie Ambeso, Nega Wuha, Wudi Gemzu | 14.74 | 11.09 | 93 | 666771(84.4%) |  |  |  |  |  |  |  |
| SIM-121 | A | N.Gondar,Alefa,Genet Guancha Silasie Godguadit, Tana Beles | 14.14 | 10.89 | 95 | 596916(84.8%) |  |  |  |  |  |  |  |
| SIM-122 | A | N.Gondar,Alefa,Genet Guancha Silasie Godguadit, Tana Beles | 18.76 | 14.32 | 137 | 875749(86.2%) |  |  |  |  |  |  |  |
| SIM-123 | A | N.Gondar,Alefa,Genet Guancha Silasie Godguadit, Tana Beles | 15.74 | 12 | 119 | 703098(80.7%) |  |  |  |  |  |  |  |
| SIM-124 | B | Awi,Jawi,Block 4,Block 4, Abat Beles | 28.68 | 23.44 | 223 | 1446999(86.8%) |  |  |  |  |  |  |  |
| SIM-125 | B | Awi,Jawi,Block 4,Block 4, Abat Beles | 34.12 | 29.6 | 250 | 1763296(86.4%) |  |  |  |  |  |  |  |
| SIM-126 | B | Awi,Jawi,Block 4,Block 4, Abat Beles | 15.5 | 11.66 | 101 | 678102(86.3%) |  |  |  |  |  |  |  |
| SIM-129 | C | N.Gondar,Metema,Diviko,Asafekari,Guang | 14.73 | 11.16 | 112 | 653992(85.9%) |  |  |  |  |  |  |  |
| SIM-130 | D | N.Gondar,Metema,Diviko,Wudi Gemzu,Wudi Gemzu | 10.07 | 6.63 | 63 | 363867(85.0%) |  |  |  |  |  |  |  |
| SIM-136 | F | N.Gondar,Metema,Wudie Ambeso, Nega Wuha, Wudi Gemzu | 8.83 | 5.65 | 54 | 265346(80.3%) |  |  |  |  |  |  |  |
| SIM-137 | F | N.Gondar,Metema,Wudie Ambeso, Nega Wuha, Wudi Gemzu | 10.07 | 6.72 | 64 | 355862(82.5%) |  |  |  |  |  |  |  |
| SIM-138 | F | N.Gondar,Metema,Wudie Ambeso, Nega Wuha, Wudi Gemzu | 19.52 | 18.4 | 164 | 877101(82.9%) |  |  |  |  |  |  |  |
| SIM-139 | A | N.Gondar,Alefa,Genet Guancha Silasie Godguadit, Tana Beles | 15.63 | 14.76 | 161 | 589453(81.4%) |  |  |  |  |  |  |  |
| SIM-140 | A | N.Gondar,Alefa,Genet Guancha Silasie Godguadit, Tana Beles | 16.4 | 12.47 | 107 | 733429(87.3%) |  |  |  |  |  |  |  |
| SIM-141 | A | N.Gondar,Alefa,Genet Guancha Silasie Godguadit, Tana Beles | 13.84 | 10.83 | 96 | 519506(82.2%) |  |  |  |  |  |  |  |
| SIM-142 | B | Awi,Jawi,Block 4,Block 4, Abat Beles | 18.51 | 14.85 | 125 | 818083(86.1%) |  |  |  |  |  |  |  |
| SIM-143 | B | Awi,Jawi,Block 4,Block 4, Abat Beles | 20.79 | 16.87 | 148 | 976283(86.9%) |  |  |  |  |  |  |  |
| SIM-144 | B | Awi,Jawi,Block 4,Block 4, Abat Beles | 21.44 | 16.81 | 145 | 1016816(88.2%) |  |  |  |  |  |  |  |
| SIM-148 | D | N.Gondar,Metema,Diviko,Wudi Gemzu,Wudi Gemzu | 19.87 | 15.91 | 127 | 1000708(88.1%) |  |  |  |  |  |  |  |
| SIM-149 | D | N.Gondar,Metema,Diviko,Wudi Gemzu,Wudi Gemzu | 10.55 | 7.57 | 80 | 356156(83.1%) |  |  |  |  |  |  |  |
| SIM-150 | D | N.Gondar,Metema,Diviko,Wudi Gemzu,Wudi Gemzu | 10.75 | 13.07 | 117 | 1325976(60.7%) |  |  |  |  |  |  |  |
| SIM-151 | E | N.Gondar, Tsegedie, Ergoye, Kisha, Angereb | 11.79 | 10.26 | 115 | 346603(79.1%) |  |  |  |  |  |  |  |
| SIM-154 | F | N.Gondar,Metema,Wudie Ambeso, Nega Wuha, Wudi Gemzu | 18.79 | 15.76 | 139 | 830043(86.2%) |  |  |  |  |  |  |  |
| SIM-155 | F | N.Gondar,Metema,Wudie Ambeso, Nega Wuha, Wudi Gemzu | 14.11 | 10.64 | 95 | 602597(85.9%) |  |  |  |  |  |  |  |
| SIM-156 | F | N.Gondar,Metema,Wudie Ambeso, Nega Wuha, Wudi Gemzu | 11.3 | 7.92 | 98 | 448006(84.1%) |  |  |  |  |  |  |  |
| SIM-157 | A | N.Gondar,Alefa,Genet Guancha Silasie Godguadit, Tana Beles | 31.78 | 32.4 | 310 | 1545531(82.8%) |  |  |  |  |  |  |  |
| SIM-158 | A | N.Gondar,Alefa,Genet Guancha Silasie Godguadit, Tana Beles | 13.89 | 10.99 | 112 | 560451(80.1%) |  |  |  |  |  |  |  |
| SIM-159 | A | N.Gondar,Alefa,Genet Guancha Silasie Godguadit, Tana Beles | 31.73 | 29.96 | 261 | 1589389(84.9%) |  |  |  |  |  |  |  |
| SIM-160 | A | N.Gondar,Alefa,Genet Guancha Silasie Godguadit, Tana Beles | 20.66 | 16.75 | 160 | 967646(87.0%) |  |  |  |  |  |  |  |
| SIM-161 | B | Awi,Jawi,Block 4,Block 4, Abat Beles | 10.32 | 7.69 | 74 | 326116(82.1%) |  |  |  |  |  |  |  |
| SIM-162 | B | Awi,Jawi,Block 4,Block 4, Abat Beles | 24.06 | 20.26 | 192 | 1196464(86.9%) |  |  |  |  |  |  |  |
| SIM-163 | B | Awi,Jawi,Block 4,Block 4, Abat Beles | 13.89 | 10.48 | 94 | 575934(85.2%) |  |  |  |  |  |  |  |
| SIM-164 | B | Awi,Jawi,Block 4,Block 4, Abat Beles | 19.37 | 15.44 | 127 | 934059(87.5%) |  |  |  |  |  |  |  |
| SIM-165 | D | N.Gondar,Metema,Diviko,Wudi Gemzu,Wudi Gemzu | 19.24 | 14.93 | 129 | 915548(87.2%) |  |  |  |  |  |  |  |
| SIM-166 | D | N.Gondar,Metema,Diviko,Wudi Gemzu,Wudi Gemzu | 46.37 | 97.46 | 798 | 768042(45.8%) |  |  |  |  |  |  |  |
| SIM-167 | D | N.Gondar,Metema,Diviko,Wudi Gemzu,Wudi Gemzu | 15.3 | 12.64 | 142 | 667046(82.2%) |  |  |  |  |  |  |  |
| SIM-168 | D | N.Gondar,Metema,Diviko,Wudi Gemzu,Wudi Gemzu | 11.07 | 10.39 | 116 | 418886(70.1%) |  |  |  |  |  |  |  |
| SIM-169 | F | N.Gondar,Metema,Wudie Ambeso, Nega Wuha, Wudi Gemzu | 35.2 | 29.64 | 258 | 1841384(87.8%) |  |  |  |  |  |  |  |
| SIM-170 | F | N.Gondar,Metema,Wudie Ambeso, Nega Wuha, Wudi Gemzu | 15.78 | 12.64 | 147 | 680018(85.9%) |  |  |  |  |  |  |  |
| SIM-171 | F | N.Gondar,Metema,Wudie Ambeso, Nega Wuha, Wudi Gemzu | 26.57 | 21.34 | 175 | 1394245(87.8%) |  |  |  |  |  |  |  |
| SIM-172 | F | N.Gondar,Metema,Wudie Ambeso, Nega Wuha, Wudi Gemzu | 38.42 | 31.97 | 264 | 2107113(88.2%) |  |  |  |  |  |  |  |
| SIM-173 | C | N.Gondar,Metema,Diviko,Asafekari,Guang | 20.94 | 16.42 | 146 | 993807(87.0%) |  |  |  |  |  |  |  |
| SIM-175 | C | N.Gondar,Metema,Diviko,Asafekari,Guang | 20.35 | 16.09 | 134 | 957661(87.9%) |  |  |  |  |  |  |  |
| SIM-176 | C | N.Gondar,Metema,Diviko,Asafekari,Guang | 21.64 | 17.11 | 164 | 1022991(88.2%) |  |  |  |  |  |  |  |
| SIM-177 | C | N.Gondar,Metema,Diviko,Asafekari,Guang | 23.39 | 23.45 | 213 | 1047508(82.2%) |  |  |  |  |  |  |  |
| SIM-178 | C | N.Gondar,Metema,Diviko,Asafekari,Guang | 19.35 | 19.46 | 180 | 1084974(79.7%) |  |  |  |  |  |  |  |
| SIM-179 | A | N.Gondar,Alefa,Genet Guancha Silasie Godguadit, Tana Beles | 6.19 | 3.14 | 53 | 110192(65.5%) |  |  |  |  |  |  |  |
| SIM-181 | A | N.Gondar,Alefa,Genet Guancha Silasie Godguadit, Tana Beles | 27 | 21.98 | 179 | 1410113(88.0%) |  |  |  |  |  |  |  |
| SIM-182 | B | Awi,Jawi,Block 4,Block 4, Abat Beles | 11.71 | 9.22 | 86 | 405978(82.7%) |  |  |  |  |  |  |  |
| SIM-183 | B | Awi,Jawi,Block 4,Block 4, Abat Beles | 25.1 | 20.07 | 182 | 1241836(88.8%) |  |  |  |  |  |  |  |
| SIM-184 | B | Awi,Jawi,Block 4,Block 4, Abat Beles | 21.9 | 17.78 | 160 | 1041584(87.3%) |  |  |  |  |  |  |  |
| SIM-185 | D | N.Gondar,Metema,Diviko,Wudi Gemzu,Wudi Gemzu | 13.38 | 10.58 | 101 | 534065(83.2%) |  |  |  |  |  |  |  |
| SIM-186 | D | N.Gondar,Metema,Diviko,Wudi Gemzu,Wudi Gemzu | 21.54 | 17.84 | 158 | 1089997(85.1%) |  |  |  |  |  |  |  |
| SIM-187 | D | N.Gondar,Metema,Diviko,Wudi Gemzu,Wudi Gemzu | 13.32 | 11.41 | 125 | 634590(79.8%) |  |  |  |  |  |  |  |
| SIM-188 | F | N.Gondar,Metema,Wudie Ambeso, Nega Wuha, Wudi Gemzu | 21.17 | 16.95 | 164 | 1002081(88.8%) |  |  |  |  |  |  |  |
| SIM-189 | F | N.Gondar,Metema,Wudie Ambeso, Nega Wuha, Wudi Gemzu | 23.94 | 19.17 | 198 | 1207823(88.3%) |  |  |  |  |  |  |  |
| SIM-190 | F | N.Gondar,Metema,Wudie Ambeso, Nega Wuha, Wudi Gemzu | 22.88 | 19.45 | 188 | 1129978(87.0%) |  |  |  |  |  |  |  |
| SIM-191 | E | N.Gondar, Tsegedie, Ergoye, Kisha, Angereb | 20.11 | 16.37 | 137 | 947855(87.0%) |  |  |  |  |  |  |  |
| SIM-192 | A | N.Gondar,Alefa,Genet Guancha Silasie Godguadit, Tana Beles | 22.38 | 18.35 | 159 | 1082001(87.9%) |  |  |  |  |  |  |  |
| SIM-193 | A | N.Gondar,Alefa,Genet Guancha Silasie Godguadit, Tana Beles | 27.18 | 22.76 | 200 | 1415018(86.7%) |  |  |  |  |  |  |  |
| SIM-204 | D | N.Gondar,Metema,Diviko,Wudi Gemzu,Wudi Gemzu | 11.18 | 7.92 | 86 | 406718(84.4%) |  |  |  |  |  |  |  |
| SIM-208 | C | N.Gondar,Metema,Diviko,Asafekari,Guang | 18.93 | 14.88 | 119 | 905136(86.1%) |  |  |  |  |  |  |  |
| SIM-210 | C | N.Gondar,Metema,Diviko,Asafekari,Guang | 60.3 | 86.45 | 538 | 567378(65.5%) |  |  |  |  |  |  |  |
| SIM-211 | D | N.Gondar,Metema,Diviko,Wudi Gemzu,Wudi Gemzu | 11.51 | 8.76 | 87 | 437338(83.3%) |  |  |  |  |  |  |  |
| SIM-212 | D | N.Gondar,Metema,Diviko,Wudi Gemzu,Wudi Gemzu | 14.9 | 11.03 | 99 | 669405(87.7%) |  |  |  |  |  |  |  |
| SIM-219 | F | N.Gondar,Metema,Wudie Ambeso, Nega Wuha, Wudi Gemzu | 14.32 | 11.13 | 101 | 596723(86.2%) |  |  |  |  |  |  |  |
| SIM-221 | F | N.Gondar,Metema,Wudie Ambeso, Nega Wuha, Wudi Gemzu | 7.83 | 5.18 | 66 | 169261(75.0%) |  |  |  |  |  |  |  |
