# Supplementary material for: Cytotaxonomic characterization and estimation of migration patterns of onchocerciasis vectors (Simulium damnosum sensu lato) in northwestern Ethiopia based on RADSeq data
Source: PLoS Negl Trop Dis. 2024 Jan 4;18(1):e0011868. doi: 10.1371/journal.pntd.0011868 (PMC10793886; doi:10.1371/journal.pntd.0011868)
Supplement: S9 Table — (DOCX) [file pntd.0011868.s010.docx]

### **Table S9.** Estimates of population genetic diversity of *Simulium damnosum sensu lato* samples collected from six locations in Ethiopia averaged across 47,638 variant sites.

| **Sample location** | **Average number of samples per locus** | **Genetic diversity (pi)** | **Number of polymorphic sites** | **Number of private alleles** | **Fis** |
| --- | --- | --- | --- | --- | --- |
| A: Selassie Godiguadit | 29.36473 | 0.07611 | 28,595 | 3,878 | 0.31313 |
| B: Block 4 | 29.14776 | 0.07643 | 28,667 | 4,083 | 0.31112 |
| C: Asakefari | 11.41265 | 0.07652 | 17,830 | 1,178 | 0.21589 |
| D: Wudi Gemzu | 24.45520 | 0.07793 | 26,562 | 2,827 | 0.30277 |
| E: Kisha | 4.64994 | 0.07649 | 10,300 | 592 | 0.13244 |
| F: Nega Wuha | 27.33778 | 0.07828 | 27,715 | 3,145 | 0.31380 |
