# Supplementary material for: Cytotaxonomic characterization and estimation of migration patterns of onchocerciasis vectors (Simulium damnosum sensu lato) in northwestern Ethiopia based on RADSeq data
Source: PLoS Negl Trop Dis. 2024 Jan 4;18(1):e0011868. doi: 10.1371/journal.pntd.0011868 (PMC10793886; doi:10.1371/journal.pntd.0011868)
Supplement: S10 Table — (DOCX) [file pntd.0011868.s011.docx]

### **Table S10.** Estimates of pairwise genetic distance (below the diagonal) and F_ST_ (above the diagonal) for six locations in Ethiopia based on 23,860 variant sites of *Simulium damnosum s.l.* collected in 6 locations in Ethiopia.

| **Sample location** | **A:** Selassie Godiguadit | **B:** Block 4 | **C:** Asakefari | **D:** Wudi Gemzu | **E:** Kisha | **F:** Nega Wuha |
| --- | --- | --- | --- | --- | --- | --- |
| A | NA | 0.0009803052 | .08235184 | .07982038 | 0.01642510 | 0.0765807633 |
| B | 0.01212146 | NA | .08951655 | .08744442 | 0.01883263 | 0.0835749616 |
| C | 0.03328864 | 0.03490083 | NA | -.00003448 | -0.00059753 | 0.02255653 |
| D | 0.02543018 | 0.02705534 | 0.02227133 | NA | 0.0001748753 | 0.02202714 |
| E | 0.03670435 | 0.03712832 | 0.04419310 | 0.03874037 | NA | 0.01808717 |
| F | 0.02420486 | 0.02565871 | 0.02124938 | 0.01399241 | 0.03741113 | NA |
