# Supplementary figures and images for: Cytotaxonomic characterization and estimation of migration patterns of onchocerciasis vectors (Simulium damnosum sensu lato) in northwestern Ethiopia based on RADSeq data
Source: PLoS Negl Trop Dis. 2024 Jan 4;18(1):e0011868. doi: 10.1371/journal.pntd.0011868 (PMC10793886; doi:10.1371/journal.pntd.0011868)

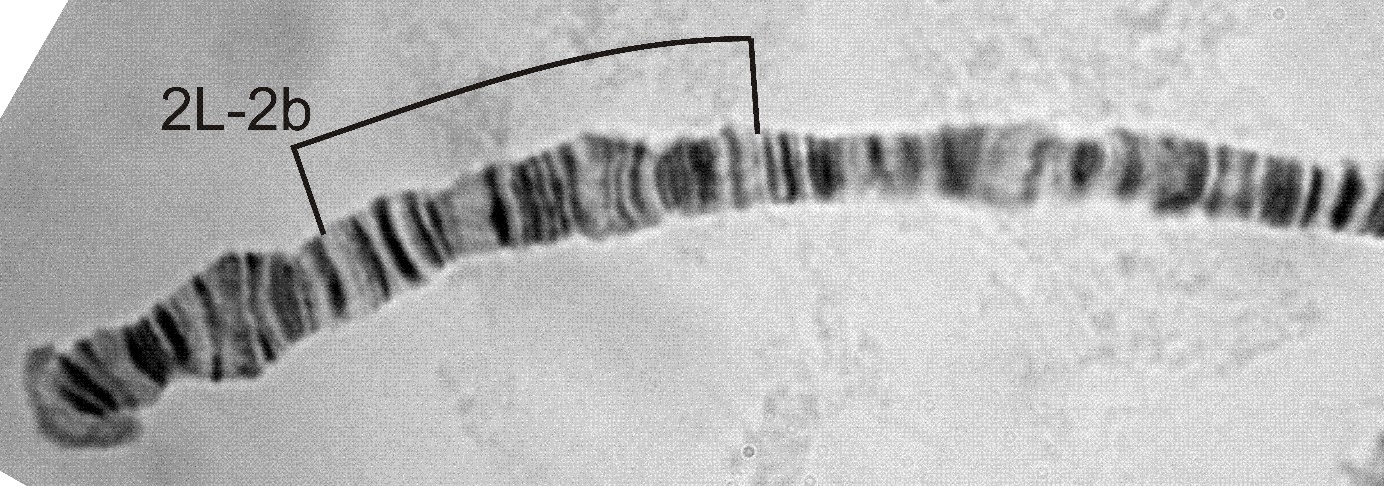


### **Fig S1.** Inversion 2L-2b marked on chromosome homozygous 2L-C.2b/C.2b.

Supplement: S1 Fig — (DOCX) [file pntd.0011868.s012.docx]

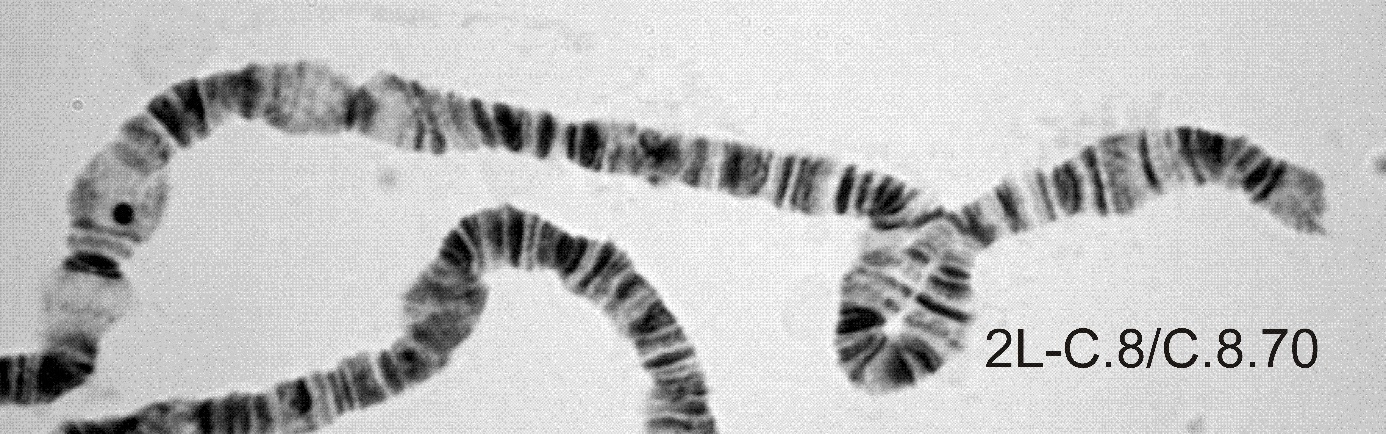


### **Fig S2.** Heterozygous inversion 2L-70.

Supplement: S2 Fig — (DOCX) [file pntd.0011868.s013.docx]

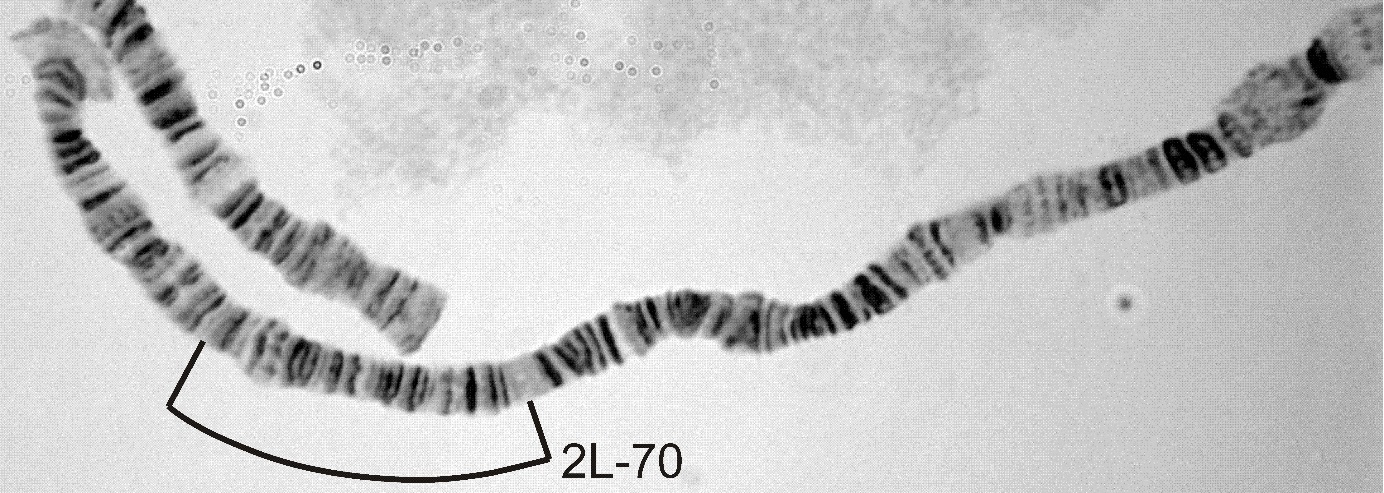


### **Fig S3.** Homozygote 2L-C.8.70/C.8.70 showing breakpoints of inversion 2L-70.

Supplement: S3 Fig — (DOCX) [file pntd.0011868.s014.docx]

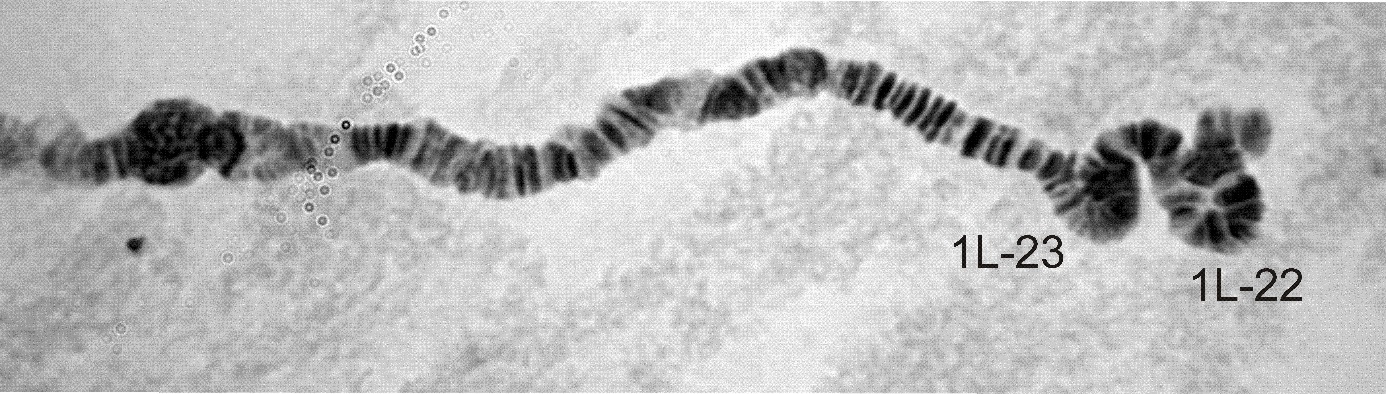


### **Fig S5.** Heterozygous inversions 1L-22 & 1L-23 on chromosome 1L-1.3/1.3.22.23.

Supplement: S5 Fig — (DOCX) [file pntd.0011868.s016.docx]

###
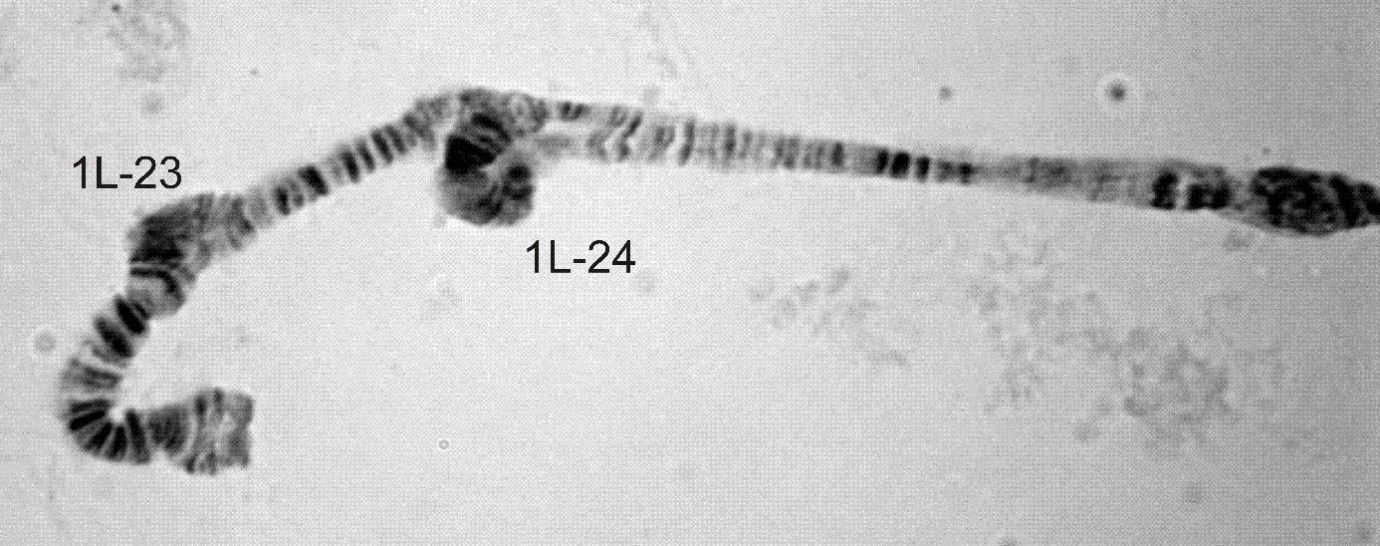


### **Fig S6.** Heterozygous inversions 1L-23 & 1L-24 on chromosome 1L-1.3/1.3.23.24.

Supplement: S6 Fig — (DOCX) [file pntd.0011868.s017.docx]
