# Supplementary material for: Cytotaxonomic characterization and estimation of migration patterns of onchocerciasis vectors (Simulium damnosum sensu lato) in northwestern Ethiopia based on RADSeq data
Source: PLoS Negl Trop Dis. 2024 Jan 4;18(1):e0011868. doi: 10.1371/journal.pntd.0011868 (PMC10793886; doi:10.1371/journal.pntd.0011868)
Supplement: S4 Fig — (DOCX) [file pntd.0011868.s015.docx]

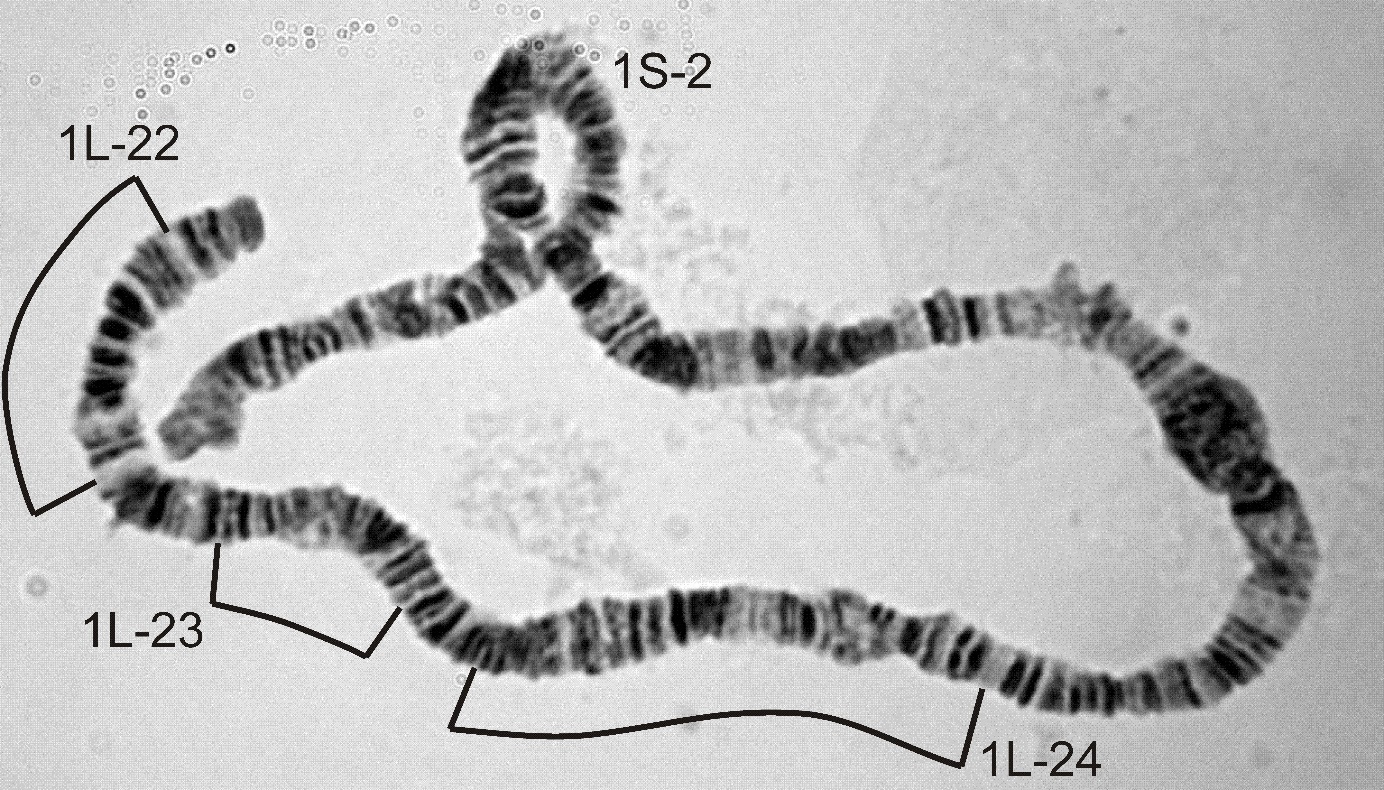


### **Fig S4.** Breakpoints of Inversions 1L-22, 1L-23 & 1L-24 on chromosome 1L-1.3/1.3. Also showing heterozygous inversion 1S-2 on chromosome 1S-2.3/3.
