# Supplementary material for: Cytotaxonomic characterization and estimation of migration patterns of onchocerciasis vectors (Simulium damnosum sensu lato) in northwestern Ethiopia based on RADSeq data
Source: PLoS Negl Trop Dis. 2024 Jan 4;18(1):e0011868. doi: 10.1371/journal.pntd.0011868 (PMC10793886; doi:10.1371/journal.pntd.0011868)
Supplement: S7 Fig — (DOCX) [file pntd.0011868.s018.docx]

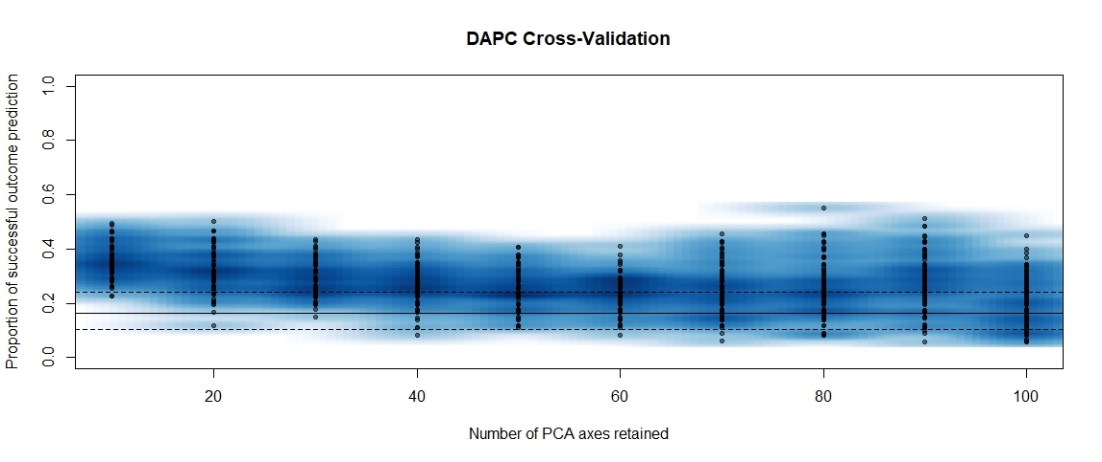


### **Fig S8.** Cross-validation for determining the optimal number of principle components to use in a discriminant analysis of principle components when K = 6, the number of sampling sites for 134 *Simulium damnosum s.l.* flies sequenced and genotyped at 23,860 variant sites in linkage equilibrium. The optimal number of PCs was inferred.
