# Supplementary material for: Cytotaxonomic characterization and estimation of migration patterns of onchocerciasis vectors (Simulium damnosum sensu lato) in northwestern Ethiopia based on RADSeq data
Source: PLoS Negl Trop Dis. 2024 Jan 4;18(1):e0011868. doi: 10.1371/journal.pntd.0011868 (PMC10793886; doi:10.1371/journal.pntd.0011868)
Supplement: S8 Fig — (DOCX) [file pntd.0011868.s019.docx]

**
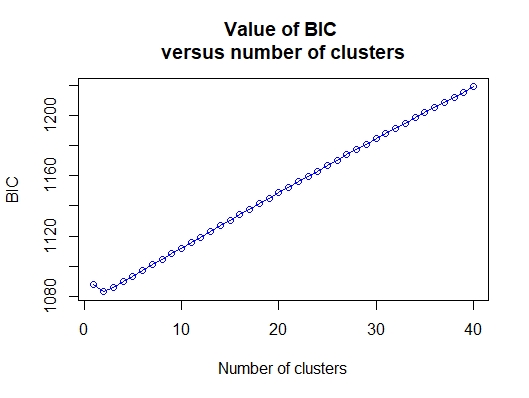
**

### **Fig S9.** Bayesian Information Criterion (BIC) for the number of clusters in nuclear sequence data of *Simulium damnosum s.l*. from Ethiopia from K = 1 through K = 40. The interpretation is that the optimal number of groups inferred in the data is K = 2.
