# Supplementary material for: Cytotaxonomic characterization and estimation of migration patterns of onchocerciasis vectors (Simulium damnosum sensu lato) in northwestern Ethiopia based on RADSeq data
Source: PLoS Negl Trop Dis. 2024 Jan 4;18(1):e0011868. doi: 10.1371/journal.pntd.0011868 (PMC10793886; doi:10.1371/journal.pntd.0011868)
Supplement: S9 Fig — (DOCX) [file pntd.0011868.s020.docx]

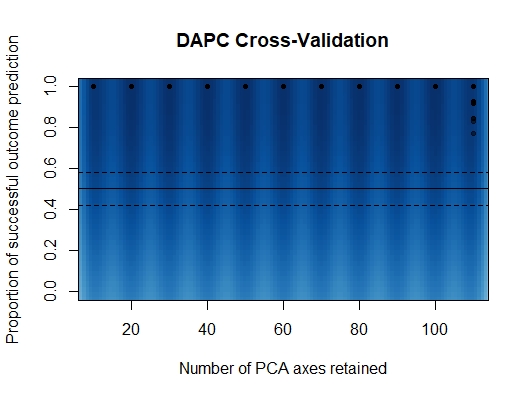


### **Fig S10.** Results of cross-validation using 100 replicates for determining the optimal number of principle components to use in a discriminant analysis of principle components when K = 2, the number of clusters inferred based on nuclear sequence data of *Simulium damnosum s.l.* flies collected in Ethiopia. The optimal number of PCs was inferred to be 90, but note that there is significant variation across the simulations.
