# Supplementary material for: Cytotaxonomic characterization and estimation of migration patterns of onchocerciasis vectors (Simulium damnosum sensu lato) in northwestern Ethiopia based on RADSeq data
Source: PLoS Negl Trop Dis. 2024 Jan 4;18(1):e0011868. doi: 10.1371/journal.pntd.0011868 (PMC10793886; doi:10.1371/journal.pntd.0011868)
Supplement: S10 Fig — (DOCX) [file pntd.0011868.s021.docx]

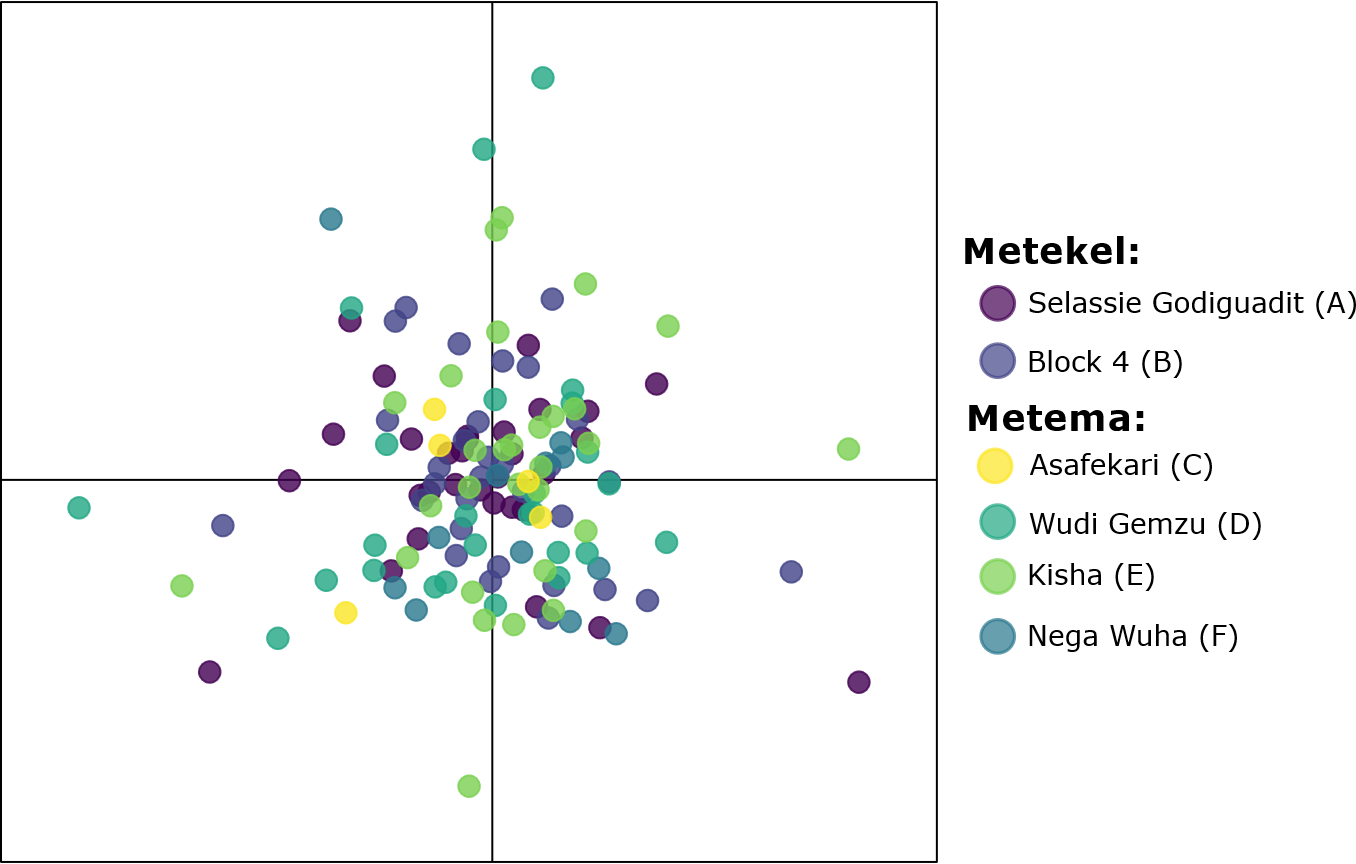


### **Fig S10.** The third (0.89% of all variance) and fourth (0.87%) principal components from analysis of 23,860 genetic variants in linkage equilibrium of *Simulium damnosum s.l.* blackflies collected at 6 sites in Ethiopia.
