# Supplementary material for: Cytotaxonomic characterization and estimation of migration patterns of onchocerciasis vectors (Simulium damnosum sensu lato) in northwestern Ethiopia based on RADSeq data
Source: PLoS Negl Trop Dis. 2024 Jan 4;18(1):e0011868. doi: 10.1371/journal.pntd.0011868 (PMC10793886; doi:10.1371/journal.pntd.0011868)
Supplement: Attachment — Submitted filename: Review.docx [file pntd.0011868.s022.docx]

The objectives were achieved, however, there are some gaps in order to understand the hypotheses proposed at the end, for example, it is not known if in the areas between the two subfocus there are possible breeding grounds for the species or if all the stream dry up.

And the other suggestion.

The discussion should go deeper with more recent articles on the genes that have been used to identify the species and especially the use of CO1 and its ability to discriminate the species, currently there is literature from other regions that address this topic.
