# Supplementary material for: Cytotaxonomic characterization and estimation of migration patterns of onchocerciasis vectors (Simulium damnosum sensu lato) in northwestern Ethiopia based on RADSeq data
Source: PLoS Negl Trop Dis. 2024 Jan 4;18(1):e0011868. doi: 10.1371/journal.pntd.0011868 (PMC10793886; doi:10.1371/journal.pntd.0011868)
Supplement: Attachment — Submitted filename: Response to referees.docx [file pntd.0011868.s023.docx]

Wofosoosjfjofjosjfo

**@LJMUofficial	@LJMU	@ljmu**

**ljmu.ac.uk**

14 Nov 2023

James Cotton	Nigel Beebe
Academic Editor	Section Editor
PLOS Neglected Tropical Diseases

RE: Manuscript PNTD-D-23-00711

Dear Drs Cotton and Beebe,

Thank you very much for the decision on our manuscript “Cytotaxonomic characterization and estimation of migration patterns of onchocerciasis vectors (*Simulium damnosum sensu lato*) in northwestern Ethiopia based on RADSeq data”. We are very pleased that the referees found this interesting, of relevance to the PLOS NTD readership, and of value to the scientific and public health community. We thank them (and you) for their helpful comments and suggestions. Below, we detail how we address these. Original editorial/review comments are given in bold to separate these from our responses.

**Firstly, apologies for the delay in returning this manuscript - its was very challenging to find reviewers for this manuscript. I'm delighted that three very well-qualified reviewers all agree on the importance and high quality of the manuscript, and it will make a very nice contribution to PLoS NTD. Reviewers #1 and #3 have very few comments, athough I note the important point made by #3 that something should be said around the ethical and regulatory framework under which human landing catches took place.**

We thank you for your efforts in finding reviewers. We are pleased to see that the referees agree on the importance and quality of our work. We address the issue of ethics concerning landing catches below.

**The second reviewer makes some very helpful and detailed feedback to improve the manuscript. Most of these are small comments on the text - and I add some tiny edits I noticed in addition to these below. The most substantial suggestions that reviewer #2 makes are suggesting some more explicitly 'spatial' genetic approaches that might add to the presentation here. These are nice ideas, and I invite the authors to at least address this in a revision, but I leave it to their expertise whether they actually attempt these analyses or if they wish to merely address in the discussion what could be done in future: in particular, I suspect that the largely clustered adult sampling sites within each focus means that these spatially explicit approaches might not add enormously to the results already presented here.**

These are dealt with in detail below, but in brief, geospatially explicit approaches might result in biased results because the sampling is highly clustered within two transmission zones, and also because it represents genetic diversity at a single time point in an organism with highly heterogeneous densities.

**Tiny editorial comments:**

**line 373: (in this case, Hardy-Weinberg equilibrium based on allele frequencies). I don't think this is quite right, as 'Hardy-Weinberg equilibrium' isn't a parameter of the structure model - its an assumption of the method that subpopulations are in HWE.**

We agree that this was not well phrased. HWE (and, depending on the model, linkage equilibrium) is used to optimize allele frequencies for each population (calculating the likelihood in a Bayesian MCMC framework). We have changed this to read “The Bayesian model-based method implemented in the program *structure* estimates the cluster membership of each individual given a certain number of possible clusters, *K* [53]. The algorithm optimizes the groupings of those individuals to meet the assumptions of Hardy-Weinberg and linkage equilibrium.”

**Line 437: from southwest in rainy season and from northeast in dry season -> "from the southwest in the rainy season and from the northeast in the dry season"**

Changed as suggested. Thank you.

**line 438 - the section on seasonal wind direction was just a tiny bit hard to follow - the message is clear once you've read it twice: that in the dry season few flies are around, so the prevailing wind from Metema -> Metekel is unlikely to lead to much migration, while the prevailing wind direction in the rainy season will lead to movement Metekel -> Metema. But I wonder if the authors can revise this to make this message a bit more explicit -**

We have attempted to clarify by altering the text to read: “The wind direction in this area is seasonal, from the southwest in the rainy season and from the northeast in the dry season. While northerly and southerly wind-assisted migration could both theoretically occur, ephemeral breeding sites affected by reduced rainfall would mean that far fewer flies would be available to be transported in the dry season than in the rainy season, when flies are abundant and winds are from the southwest. Such northerly migration could therefore result in long-distance migration from Metekel to Metema.”

Our further responses to reviewers are detailed below.

Sincerely,

Dr Craig Wilding

Reader in Evolutionary Genetics and Programme Leader for Biology

School of Biological and Environmental Sciences

Liverpool John Moores University

Room 230 James Parsons Building

Byrom Street

Liverpool

L3 3AF

**Reviewer #1: The manuscript uses two approaches to analyse the population structure of onchocerciasis vectors in Ethiopean foci. Importantly, the authors include a excellent polytene chromosome analysis. Genome syteny as revealed by polytene chromosome cytogenetics forms the basis of species-level taxonomy in Simuliidae and without itthe literature can become clouded by taxonomically unplaced "genotypes" of unknown affinity. The second prong of this paper, RAD-Seq analysis, has been successfully used in other vector taxa, e.g., mosquitoes. The authors use this newer technique to great effect, demonstrating not only 2 geneetically isolated entitites in the local damnosum s.l. population but also the technique's utility for migtration and re-colonization studies that will be critical for elimination efforts.**

**Both approaches were carefullly done and well analysied.**

We thank the reviewer for this kind response and for their efforts in reviewing our manuscript.

**Reviewer #2: General points:**

**The authors use PCA and STRUCTURE output as the basis for inferring possible long-distance migration between two river basins (previously known to be the basis of Simulium population clustering). The Simulium blackfly is an understudied organism and there is much left to learn about its ecology and genetics - so I was really happy to read this manuscript. This manuscript identifies some interesting signatures of population structure, and potential long-distance migration between two different transmission zones that supports existing genomic work using parasite mitochondrial genome data by one of the groups involved suggesting that blackflies, and Onchocerca parasites, are structured by river basin. As such, it is a useful addition to the field. This is a really interesting dataset, and I felt that there are ample opportunities for more in-depth and informative analyses of these data that would be informative for detecting the extent to which variation in dispersal, and inversion polymorphism frequency, contributes to the signal observed here. The authors are careful to acknowledge the limitations of their study, and couch their claims accordingly.**

We thank the reviewer for these comments and appreciate the time and consideration they have put into assessing and improving our manuscript.

**In the absence of further analysis (which I think they could do, and would add substantially to the paper), my main comment is that I feel it could be more grounded in the extensive body of literature that uses genetic data to understand organism movement, as well as the contribution of spatial structure and inversion polymorphism to observed patterns of genetic structure. I think this would add a lot of interesting detail to the background and interpretation of the findings.**

**e.g.**

**https://urldefense.com/v3/__https://www.biorxiv.org/content/10.1101/2020.03.01.971333v3.full__;!!IhKztkE!Z-eoszRSNBRDseUj-qfrX4swzrM6ZkYM-e9dMHby0HhxegtCrDXWihdUFoWowYBpLwxjDQsXWC2IKxdJVRVaEA$**

**https://urldefense.com/v3/__https://pubmed.ncbi.nlm.nih.gov/36524932/__;!!IhKztkE!Z-eoszRSNBRDseUj-qfrX4swzrM6ZkYM-e9dMHby0HhxegtCrDXWihdUFoWowYBpLwxjDQsXWC2IKxelnWOYpw$ (and many others like it)**

**https://urldefense.com/v3/__https://onlinelibrary.wiley.com/doi/full/10.1111/mec.15707__;!!IhKztkE!Z-eoszRSNBRDseUj-qfrX4swzrM6ZkYM-e9dMHby0HhxegtCrDXWihdUFoWowYBpLwxjDQsXWC2IKxcmOvQQAg$**

**https://urldefense.com/v3/__https://academic.oup.com/genetics/article/215/1/193/5930485?login=true__;!!IhKztkE!Z-eoszRSNBRDseUj-qfrX4swzrM6ZkYM-e9dMHby0HhxegtCrDXWihdUFoWowYBpLwxjDQsXWC2IKxeqGvTZFA$**

**https://urldefense.com/v3/__https://www.annualreviews.org/doi/abs/10.1146/annurev-ecolsys-110316-022659__;!!IhKztkE!Z-eoszRSNBRDseUj-qfrX4swzrM6ZkYM-e9dMHby0HhxegtCrDXWihdUFoWowYBpLwxjDQsXWC2IKxcZLFvuvA$**

We agree that inversion polymorphisms are likely to be an important contributor to genetic structure in blackflies, given the frequency with which inversions occur and their observation in the northwest Ethiopia. One of the challenges faced by our study is that there is currently no published genome of any *Simulium* species, and thus the RADSeq data cannot be used to explicitly identify or even speculate on how inversions contribute to the observed differentiation. The analysis presented by e.g., Huang et al 2020 are possible because, while their data were also generated using RADSeq, they had a genome to orient the resulting sequenced genome fragments. We did attempt to explore whether there were particular contigs where differentiation was concentrated (not shown), but the contigs are simply too short (135 bp) and differentiation too diffuse across contigs to observe any significant associations between sequence fragments.

**I have never performed, analysed any data from, or been involved in, cytogenetic analyses, so I cannot comment on the methods and results for these sections of the paper.**

**Specific comments below:**

**Line 86: Do the authors have a citation for this statement?**

We have added a citation here as recommended.

**Line 87/88: There is a lot of theoretical and practical work supporting this in populations generally that could be cited (e.g. Ilkka Hanskki’s work in Glanville Fritillaries, Janet Midega’s 2012 paper on mosquito larval sites and malaria case)**

In this part of the manuscript, we are not referring to arthropod movement in general, but to the impact of vector movement on the spread of disease. To date, it does not appear that anyone has identified migratory *infective* vectors. We have added multiple citations for migration of mosquitos, in addition to those already cited with regards to blackflies, as recommended.

**Line 216 (Fig 1). The map is quite grainy - please could a higher-definition map be provided?**

We believe that the graininess is an artefact of the automated conversion to PDF. The submitted .tif file was clear and of high resolution.

**Line 254: The authors haven’t pruned their data for linkage disequilibrium (LD). LD can mask signals of population structure, or create spurious signatures of the population subdivision, especially when inversions are concerned. I suggest the authors at least examine the effect of LD pruning on their PCA to see the extent to which pruning for LD changes the PCA result, and if it does, do this for the STRUCTURE analysis too.**

**Informative blog posts here: https://urldefense.com/v3/__https://privefl.github.io/bigsnpr/articles/how-to-PCA.html__;!!IhKztkE!Z-eoszRSNBRDseUj-qfrX4swzrM6ZkYM-e9dMHby0HhxegtCrDXWihdUFoWowYBpLwxjDQsXWC2IKxeyV98CRg$ . https://urldefense.com/v3/__http://alimanfoo.github.io/2015/09/28/fast-pca.html__;!!IhKztkE!Z-eoszRSNBRDseUj-qfrX4swzrM6ZkYM-e9dMHby0HhxegtCrDXWihdUFoWowYBpLwxjDQsXWC2IKxf2q34ckA$**

We agree that LD can be a problem for inferring geographic population structure, and to test the potential impact of LD on our results, have pruned our data considering LD (with a highly conservative cut-off of r2>0.1) and re-ran the PCA and DAPC analyses. Removing sites that might be in LD did not qualitatively impact any of the results. Note that one of the challenges with this approach in general is that population structure can be the cause of LD (in addition to selection or inversions), and thus without information about where sites in LD are located physically on the chromosomes, we cannot discriminate between reasons for observed LD. This lack of a genome (of any *Simulium* species) motivated our reasoning for originally not excluding sites in LD. Since the results were not substantially different, we have replaced Fig 3 with these nearly identical results, and do not discuss the LD results in detail in the main text.

**Line 354: I find the PCAs in 3A and B quite difficult to interpret as the plots are overcrowded with lines and ellipses. I appreciate for DAPC this is part of the analysis and interpretation but for ease of interpretation I would include at least the PCA in Fig 3A without lines or ellipses / move the annotated standard PCA to supplementary.**

**It’s also not clear whether A and B are both DAPC output or not, as ‘standard’ PCA generally doesn’t include lines/ellipses - if Fig 3A is not DAPC, where do the lines and ellipses come from?**

The intent of ellipses in a PCA is to clarify the extent of overlap between different populations (i.e., the same reason that the ellipses are included in DAPC plots). However, we have removed these from Fig 3 as requested.

**It would assist with interpretation of the results if the authors could include PCA results from lower PCs (3,4 and 5,6), as well as the amount of variation in the data explained by each PC.**

**The lower PC’s could go into supplementary information. This would allow readers to examine the extent to which the PCAs represent variation in the data and also interpret subtle structure often revealed by examining lower PCs.**

The lower PCs are not informative and have been added to supplemental materials as recommended (Supplemental File S1 Fig S10).

**Line 417: The three groups in the Metekel cluster on the Y axis of Fig 3A look like they could, possibly, be caused by an inversion polymorphism. See, amongst many others:**

**https://urldefense.com/v3/__https://onlinelibrary.wiley.com/doi/10.1111/mec.15428__;!!IhKztkE!Z-eoszRSNBRDseUj-qfrX4swzrM6ZkYM-e9dMHby0HhxegtCrDXWihdUFoWowYBpLwxjDQsXWC2IKxf6Gd2G-w$ - hard to tell with these data without them being anchored by chromosome, but sometimes inversions can drive very strong signal in PCA. LD pruning would help resolve whether this is the case.**

While this would be an intriguing result, we are not convinced that the groupings along PC2 are informative about reproductive isolation in the absence of other data, particularly data on how the observed variation is distributed within the genome.

**Line 419: I would be very interested to see how distinct the two clusters are. Examining lower PCs, and quantifying % of variance explained by each PC, as well as quantifying Fst between the different sites and clusters (presented as a heatmap of pairwise between-site Fst), would be informative in this case and easy to achieve with the data and workflows the authors possess. Population structure may vary across the genome, and geographic variation in this can bias analyses such as PCA (inversion polymorphisms are a great example of that - see https://urldefense.com/v3/__https://pubmed.ncbi.nlm.nih.gov/30459280/__;!!IhKztkE!Z-eoszRSNBRDseUj-qfrX4swzrM6ZkYM-e9dMHby0HhxegtCrDXWihdUFoWowYBpLwxjDQsXWC2IKxdTUBqj-A$ , and the Anopheles literature) to give clustering patterns that reflect inversion frequency as opposed to neutral genetic structure. Certainly when genome-wide data are examined over RAD-Seq / cytogenetic data, previously defined population subdivisions break down in favour of regions of the genome where gene flow is restricted.**

We agree that inversion polymorphisms are likely to be very important in blackflies, given the cytogenetic data on the frequency with which they occur, but are not able to explore this without a genome assembly. Our concern is that we do not wish to imply that we have inversion polymorphism data for the blackflies that we have sequenced, and as a result are attempting to avoid speculative discussion. However, we have added the following “While we do not have cytogenetic data from blackflies from either genetic cluster, reproductive isolation has been shown to evolve in other dipterans as a result of chromosome inversions, and inversions have contributed to adaptive variation for traits such as preference for indoors versus outdoors, optimal climate, or insecticide resistance in mosquitos (e.g., [63-65]).” [beginning line 435]

As recommended, we have provided the % variance explained by each PC in the legend of Fig 3, and the plots of lower PCs in Fig S10.

We have calculated F_ST_ and pairwise genetic distance (Supplemental File S1 Table S11), and have added the following text to Supplemental File S1:

“Isolation-by-distance occurs when genetic variation of a continuously distributed species with low dispersal range is associated with geographic distance rather than differentiation arising as a result of discontinuous geographic isolation or other types of reproductive isolation. We performed a statistical comparison of geographic distance between sampling sites, estimated using *geosphere* v. 1.5.18 (<https://github.com/rspatial/geosphere>) with pairwise genetic distance and with F_St_, calculated using the R package *hierfstat* v 0.5.11 (Table S10), using a Monte-Carlo Mantel test with 10 million permutations using *ade4* v 1.7.22. The result of the permutations indicated that the data were not consistent with an isolation-by-distance model (genetic distance: p = 0.1263583, F_ST_: p = 0.0499657). Note that the fundamental assumption behind this test is that the data are from a single species. For our dataset, we could not ascertain whether the two clusters represented different samples of the same species, or two different species, and thus the biological interpretation of these results is unclear.”

**Line 417 - again:**

**The authors are careful to equivocate between different demographic scenarios underlying the clustering pattern, and appearance samples from both clusters in one of the sampling sites. If the authors wished, they could analyse their data further to identify whether one of their two proposed scenarios (discrete structure with recent migrants, or continuous structure with two distinct, non-interbreeding populations) are more likely. STRUCTURE assigns populations in the presence of spatial autocorrelation in relatedness between geographically close samples. Perhaps blackflies exhibit strong signals of isolation-by-distance, which would account for two populations, sampled far apart, appearing to be from discrete transmission zones/river basins, when actually they are part of a continuously structured population (reflected in the mitochondrial data, which from a relatively short marker and therefore less prone to signatures of isolation-by-distance as will have fewer segregating**

**sites). A test for isolation-by-distance (from kinship, or sitewise Fst) would inform the extent to which spatial structure may be contributing to the clustering pattern in the STRUCTURE output and the extent to which these clusters are actually discrete populations, and then allow for more robust conclusions to be drawn about potential migration between discrete populations - and whether the data are likely to support either of the hypotheses presented in Line 454. The conSTRUCT software package by Gideon Bradburd aims to parse between similar scenarios:** [**https://urldefense.com/v3/__https://academic.oup.com/genetics/article/210/1/33/6088031__;!!IhKztkE!Z-eoszRSNBRDseUj-qfrX4swzrM6ZkYM-e9dMHby0HhxegtCrDXWihdUFoWowYBpLwxjDQsXWC2IKxeyEbc9TQ$**](https://urldefense.com/v3/__https://academic.oup.com/genetics/article/210/1/33/6088031__;!!IhKztkE!Z-eoszRSNBRDseUj-qfrX4swzrM6ZkYM-e9dMHby0HhxegtCrDXWihdUFoWowYBpLwxjDQsXWC2IKxeyEbc9TQ$)

We agree that IBD is an important characteristic to test when exploring whether there are geographically distinct breaks in populations. The data are not consistent with IBD; we have added this result to the supplemental materials as described above. Given the relatively restricted sampling of our work, and because of the uncertainty about the reproductive isolation between the inferred two genetic clusters of blackflies, we used a simple Mantel test rather than a model-based approach, and present these results in Supplemental File S1 rather than in the main text.

Please note that the key finding we present is not that there are discrete populations, but that these analyses have detected gene flow between two onchocerciasis endemic regions that are being managed independently. Because this has potential implications for onchocerciasis management, whether this is a continuous population (stepping stone model, given what we know about *Simulium* breeding sites) or two very discrete populations with rare gene flow (i.e., wind-assisted migration), is important to discriminate, but would require improved sampling from within the “buffer” zone.

**Perhaps the two (though I actually see four) clusters in the PCA and STRUCTURE are due to geographic variation in inversion polymorphism frequency (e.g. the 3 clusters we see along the y axis of 3A is due to 0/0, 1/0, 1/1 of one inversion, and the single cluster from Metema is 0/0, 1/1 for a different segregating inversion), and individuals with an inversion karyotype more typical for Metekel are present in Metema but at a slightly lower frequency? Examining the effect of LD pruning as suggested above will be informative for this.**

Unfortunately, we cannot do an analysis identifying the impacts of an inversion, as each contig is only 135 bp long and we cannot infer how they are connected. Measures of site-based genetic differentiation, such as pairwise F_ST_, between the two clusters of blackflies did not easily identify what could be hypothesized to be a contiguous block of high-F_ST_ SNPs as would be anticipated if an inversion were driving differentiation (not shown).

Note that the “clusters” observed in the PCA are not segregating by collection site. We agree that there are a number of very interesting evolutionary questions that remain about *Simulium* speciation and genetic differentiation, and that these will be worthwhile to pursue. Genomes produced via long-read sequencing would greatly assist with these kinds of analyses. However, we need to be circumspect and avoid over-interpreting the results we are presenting in this particular manuscript, and our intent for this manuscript is to keep the focus on the implications for onchocerciasis management.

**Assigning directionality to these types of data (e.g. “northward migration” is very difficult. There are methods enabling this, for example BayesAss or DisperseNN, if the authors wished to find directionality of migration.**

The evidence presented here indicates that there is a genetically distinct cluster of blackflies found predominantly in the south and only rarely in the north. This would be a stronger indication of northward movement, consistent with knowledge about seasonal wind direction. However, without sampling from within the buffer zone, inferring directionality could be problematic.

**Line 459: Could there have been contamination or sequencing error?**

This is of course possible. We have amended the text to read “When incongruence is observed, it can be attributed to biological processes — such as incomplete lineage sorting (when shared ancestral polymorphism is randomly lost in one population) or introgression (interspecific hybridization followed by unidirectional backcrossing) — or to systematic errors in phylogenetic reconstruction, or even to PCR/sequencing error.”

**Line 496: “Limitations of the study” - line left in?**

Thank you; this line has been removed.

**Reviewer #3: the objectives are clear and articulated with the research question**

**The design is very appropriate and introduce one more approach to identify the species**

**The population is described clearly**

We thank the reviewer for their remarks and for the effort put into reviewing our work.

**Due to the difficulty in finding suitable stages for identification by cytotaxonomy, this approach remains unresolved, but the authors discuss it very well.**

**There is not a clear description about ethical requirements, taking into account the authors catched insects landing on human, but they did not explain or described if there are some permission? in Ethiopia are some requirements? Which institution approved this work?**

We now include the following statement in the Methods section (under *Collection of anthropophilic adult female blackflies*):

“Ethical approval was obtained from the Ethiopian Public Health Institute (EPHI) and local administrative offices prior to collection. All staff involved in collections received standard training, consented to undertake fly collections, and received ivermectin as part of the routine MDA.”

**--------------------**

**Results**

**Reviewer #1: The results of both approaches are complementary and extremely significant. Both the cytogenomic (polytene chromosome) and RAD-Seq will be valuable for the epidemiology of onchocerciasis in the region, and for control/monitoring efforts. The results are well illustrated, and the figures enhance the reporting of the results. The revealing of a new cytoform (potentially a new species) in the damnosum complex is significant, as is the RAD-Seq demonstration of two non-interbreeding forms.**

**I was intrigued by the discovery of a the polymorphic inversion 2L.8, since it parallels the multiple roles of the IIIL-19 inversion in the Simulium vernum complex, which can likewise be fixed autosomal, sex-linked or autosomal polymorphic.**

A comprehensive metadata analysis of chromosome evolution in *Simulium* would be very interesting.

**The ambiguity/complexity in the mtDNA bar-coding is also an important result. There is**

**Reviewer #2: (No Response)**

**Reviewer #3: Yes, the analysis and the figures are clear**

**--------------------**

**Conclusions**

**Reviewer #1: The conclusions are well justified by the data. The deomonstration of a new cytoform/species in the damnosum complex and two non-interbreeding forms in the Ethiopean foci is important and timely.**

**Reviewer #2: (No Response)**

**Reviewer #3: The authors discuss the biases found and put forward two possible hypotheses that may respond to what they found.**

**yes, they highlight the importance for public health, especially the possible influence on the onchocerciasis elimination program.**

**--------------------**

**Summary and General Comments**

**Reviewer #1: In view of the renewed efforts to control and hopefully eliminate onchocerciasis in much of sub-Saharan Africa, this paper is both impportant and timely. It will aid in design of control efforts, not only in the Ethiopean foci but elsewhere by highlighting important vector population considerations. It will also help avoid reliance on fast but dubious bar-coding methods by the paper's demonstration of the complexity of mitochondrial sequence variant distribution within the complex.**

**Both the bar-coding and polyteene results contribute to the "pure science" understanding of introgression and sharing of polymorphisms.**

**Reviewer #2: (No Response)**

**Reviewer #3: there are some gaps in order to understand the hypotheses proposed at the end, for example, it is not known if in the areas between the two subfocus there are possible breeding grounds for the species or if all the stream dry up.**

We agree that a more specific geospatial approach that takes into account how the suitability of water bodies change seasonally would be an interesting area for future exploration.

**And the other suggestion.**

**The discussion should go deeper with more recent articles on the genes that have been used to identify the species and especially the use of CO1 and its ability to discriminate the species, currently there is literature from other regions that address this topic.**

In general, when COI is compared to nuclear or cytotaxonomic data in *Simulium*, the taxonomic delimitation inferred does not match. We focus most of our discussion on this in the introduction, lines 163-168, and refer to this again in lines 474-476. We would not recommend using COI as a barcoding gene in *Simulium*.
